# Invasive *Staphylococcus epidermidis* uses a unique processive wall teichoic acid glycosyltransferase to evade immune recognition

**DOI:** 10.1126/sciadv.adj2641

**Published:** 2023-11-24

**Authors:** Yinglan Guo, Xin Du, Janes Krusche, Christian Beck, Sara Ali, Axel Walter, Volker Winstel, Christoph Mayer, Jeroen D. C. Codée, Andreas Peschel, Thilo Stehle

**Affiliations:** ^1^ Interfaculty Institute of Biochemistry, University of Tübingen, Tübingen, Germany.; ^2^ Cluster of Excellence “Controlling Microbes to Fight Infections (CMFI)”, University of Tübingen, Tübingen, Germany.; ^3^ Interfaculty Institute of Microbiology and Infection Medicine Tübingen, Infection Biology, University of Tübingen, Tübingen, Germany.; ^4^ German Centre for Infection Research (DZIF), Partner Site Tübingen, Tübingen, Germany.; ^5^ Leiden Institute of Chemistry, Leiden University, Leiden, Netherlands.; ^6^ Interfaculty Institute of Microbiology and Infection Medicine Tübingen, Organismic Interactions/Glycobiology, University of Tübingen, Tübingen, Germany.

## Abstract

*Staphylococcus epidermidis* expresses glycerol phosphate wall teichoic acid (WTA), but some health care–associated methicillin-resistant *S. epidermidis* (HA-MRSE) clones produce a second, ribitol phosphate (RboP) WTA, resembling that of the aggressive pathogen *Staphylococcus aureus*. RboP-WTA promotes HA-MRSE persistence and virulence in bloodstream infections. We report here that the TarM enzyme of HA-MRSE [TarM(Se)] glycosylates RboP-WTA with glucose, instead of *N*-acetylglucosamine (GlcNAc) by TarM(Sa) in *S. aureus*. Replacement of GlcNAc with glucose in RboP-WTA impairs HA-MRSE detection by human immunoglobulin G, which may contribute to the immune-evasion capacities of many invasive *S. epidermidis*. Crystal structures of complexes with uridine diphosphate glucose (UDP-glucose), and with UDP and glycosylated poly(RboP), reveal the binding mode and glycosylation mechanism of this enzyme and explain why TarM(Se) and TarM(Sa) link different sugars to poly(RboP). These structural data provide evidence that TarM(Se) is a processive WTA glycosyltransferase. Our study will support the targeted inhibition of TarM enzymes, and the development of RboP-WTA targeting vaccines and phage therapies.

## Teaser

Opportunistic pathogen *Staphylococcus epidermidis* escapes from the host immune system by altering a major surface antigen.

## INTRODUCTION

*Staphylococcus epidermidis*, a member of coagulase-negative staphylococci, is the most frequently isolated Gram-positive bacterium from the skin and mucous membranes of all humans (*1*, *2*). The differences in skin features (thickness, folds, lipid content, densities of hair follicles, and glands) define the habitats of a large number of clonal lineages (*3*, *4*), as well as age-related dynamics of colonization (*5*). *S. epidermidis* lineages colonize the skin of virtually every human as commensals (*6*, *7*), maintaining the commonly benign relationship with their host. For instance, the resident *S. epidermidis* is necessary for optimal skin immune fitness (*8*, *9*). Many *S. epidermidis* isolates can stimulate nasal epithelia to produce antimicrobial peptides, killing pathogenic competitors (*10*), and Esp-secreting *S. epidermidis* strains are able to inhibit biofilm formation and nasal colonization of *Staphylococcus aureus* (*11*), an aggressive pathogen that causes life-threatening infections in humans (*12*, *13*).

In recent decades, however, some *S. epidermidis* clones have emerged as a major cause of hospital-acquired infections, including bloodstream infections and infections of indwelling medical devices, such as central intravenous catheters, prosthetic joint, vascular grafts, surgical site, central nervous system shunt, and cardiac devices (*1*, *14*, *15*). A major percentage of invasive *S. epidermidis* clones displays resistance to methicillin and other antibiotics, which poses a substantial clinical burden due to broad and severe treatment difficulties (*5*, *16*, *17*, *18*). The majority of such infections are caused by specific health care–associated methicillin-resistant *S. epidermidis* (HA-MRSE) lineages, several of which are usually not found on typical areas of human skin such as those of the arms or on nasal mucous membranes. The term “invasive” refers to the dominance of HA-MRSE clones in infections of sterile tissues. However, even such clones are less virulent than typical *S. aureus* clones, and their capacity to cause infections depends on contamination of indwelling medical devices such as those described above. While many of the nosocomial *S. epidermidis* clones have strong capacities to form biofilms on artificial surfaces that protect them from antibiotics and host defenses (*19*), the ST10, ST23, and ST87 clones are poor biofilm formers but alter their surfaces in a way that promotes their invasiveness (*20*). The global spread of the most prominent of these clones, ST23, has been documented recently (*18*). These clones produce an additional, *S. aureus*–type wall teichoic acid (WTA), a glycopolymer governing interactions with host cell receptors, immune effectors, and bacteriophages (*21*).

WTA is the most abundant peptidoglycan-linked glycopolymer presented on the cell surface of most Bacillota [formerly known as Firmicutes (*22*)], serving essential functions in cell wall integrity, susceptibility to bacteriophages, and resistance to antimicrobial molecules and host proteins (*21*, *23*). *S. epidermidis* usually produces glycerol 3-phosphate (GroP) WTA, which is modified with d-alanine and variable sugar residues. In contrast, most clones of the aggressive pathogen *S. aureus* express d-ribitol 5-phosphate (RboP) WTA, which is modified by d-alanine and *N*-acetylglucosamine (GlcNAc) (*21*, *23*). GlcNAc can be linked to the RboP repeating units in three different ways, which shape host and phage interactions differently (*24*). The housekeeping glycosyltransferase TarS catalyzes the β-O-GlcNAcylation of the poly(RboP) backbone at the C4 position (*25*, *26*). Some *S. aureus* clones also encode TarM, which modifies WTA in the same position, albeit with α-O-GlcNAc (*27*). Recently, we identified a third glycosyltransferase, TarP, which is encoded on a prophage in some *S. aureus* clones, and which is responsible for C3-β-O-GlcNAcylation (*28*). WTA glycosylation with GlcNAc is essential for the *S. aureus* host colonization capacities (*29*). The type of RboP-WTA GlcNAc linkages shapes the immunogenicity and interaction with certain groups of bacteriophages (*24*).

The HA-MRSE clones, ST10, ST23, and ST87, produce in addition to GroP-WTA, a second, RboP-WTA using the *tarIJLM2* gene cluster (*20*). This cluster encodes TarI, TarJ, and TarL enzymes that assemble the poly(RboP) backbone, as well as a WTA glycosyltransferase, TarM. These genes are closely related to the corresponding genes in *S. aureus* and have probably been acquired by horizontal gene transfer. The production of RboP-WTA impairs *S. epidermidis* nasal colonization but promotes persistence in the bloodstream, leading to increased mortality in a mouse sepsis model (*20*). Thus, RboP-WTA can alter the lifestyle of *S. epidermidis* from commensal to pathogenic and enable *S. epidermidis* to exchange DNA with *S. aureus* via siphoviruses that bind to RboP-WTA and are major vehicles for horizontal gene transfer in staphylococci (*20*, *30*, *31*).

Here, we report that TarM of *S. epidermidis* [TarM(Se)] incorporates glucose, instead of GlcNAc into RboP-WTA, which disables *S. aureus*–specific human immunoglobulin G (IgG) to detect *S. epidermidis* and is probably used by many HA-MRSE to remain partially undetectable in the bloodstream. Extensive structural characterization of TarM(Se)_G117R_ with donor and acceptor substrates, in particular, with product uridine diphosphate (UDP) and glycosylated poly(RboP) explains the binding mode of poly(RboP) and the catalytic mechanism of a retaining WTA glycosyltransferase. Moreover, they provide an explanation for the enzymatic differences between TarM(Se) and the corresponding *S. aureus* enzyme TarM(Sa). Our structures demonstrate that TarM(Se) is a processive WTA glycosyltransferase and provide an excellent basis for the development of TarM inhibitors that could help to impede the virulence and immune-evasion capacities of many HA-MRSE and of methicillin-resistant *S. aureus* (MRSA) clones.

## RESULTS

### RboP-WTA synthesized by the *S. epidermidis tarIJLM2* cluster differs in its antigenic properties from those of RboP-WTA from *S. aureus*

**Fig. 1.**
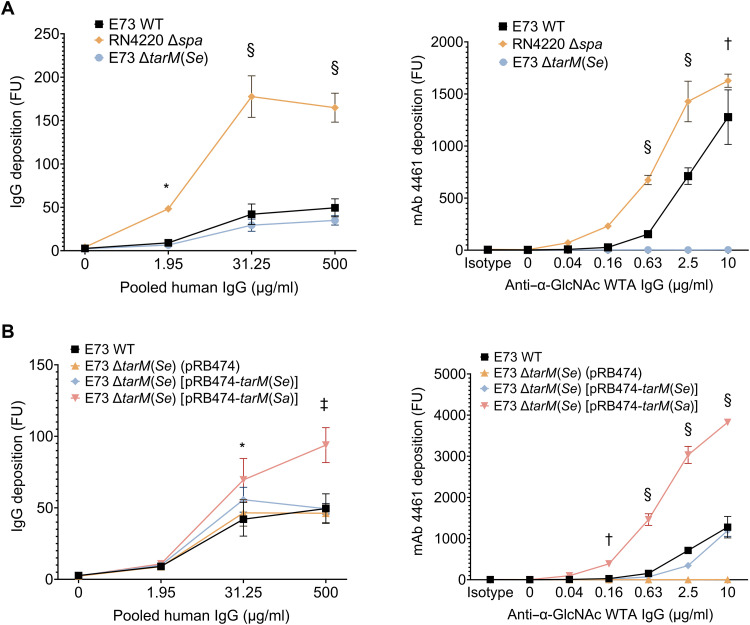
TarM(Se) decreases the binding of IgG to *S. epidermidis* or *S. aureus* with RboP-WTA. (**A**) *S. aureus* RN4220 binds much higher amounts of IgG from pooled human serum or of monoclonal IgG1 (mAb 4461) directed against RboP-WTA with α-GlcNAc than *S. epidermidis* E73. Inactivation of *tarM*(*Se*) does not further reduce pooled IgG binding to E73. (**B**) Complementation of E73 ∆*tarM*(*Se*) with *tarM*(*Sa*) leads to much higher IgG binding than complementation with *tarM*(*Se*). FU, fluorescence units. The data represent the mean ± SEM of at least three independent experiments (three biological replicates). Two-way analysis of variance (ANOVA) was used to determine statistical significance. *P < 0.05, †*P* < 0.01, ‡*P* < 0.001, and §*P* < 0.0001, difference versus *S. epidermidis* E73 wild type, as calculated by a two-way ANOVA test.

The *tarM*(*Se*) gene encodes a protein, TarM(Se), with 83% sequence similarity to the *S. aureus* TarM(Sa), which has been shown earlier to catalyze the α-O-GlcNAcylation of RboP-WTA backbone at C4 position using UDP-GlcNAc as donor substrate (fig. S1) (*27*, *32*, *33*). *S. epidermidis* E73, a clinical isolate that harbors the *tarIJLM2* gene cluster, was therefore assumed to produce the same type of glycosylated WTA as *S. aureus* strains with *tarM*(*Sa*). Although most *S. aureus* also carry the *tarS* gene, TarM(Sa) has been shown to be dominant over TarS leading to RboP-WTA, which is α-glycosylated with GlcNAc (*34*). Since WTA is a major surface antigen of *S. aureus* and the WTA GlcNAc residues are essential components of the antigenic epitope (*28*, *35*), we compared the binding of human IgG to *S. aureus* RN4220 and *S. epidermidis* E73 with or without *tarM*(*Se*) (Fig. 1A). Human IgG pooled from several healthy donors was used as virtually every human has abundant anti–*S. aureus* IgG antibodies as a consequence of previous *S. aureus* infections (*36*). *tarM*(*Se*), the last gene of the *tarIJLM2* operon, was deleted in E73, yielding mutant E73 ∆*tarM*(*Se*) with unaltered growth behavior, biofilm formation, or amount of WTA (fig. S2).

The E73 wild-type strain bound substantially lower amounts of human IgG than RN4220, a *tarM*(*Sa*)-expressing *S. aureus* strain (Fig. 1A). Note that an RN4220 mutant lacking protein A (Spa), which binds IgG unspecifically via the Fc part (*37*), was used to monitor only antigen-specific IgG binding. While a moderate difference was expected because of differences between *S. aureus* and *S. epidermidis* surface protein antigens, the difference in IgG binding was much more substantial (Fig. 1A), suggesting that the dominant WTA antigen epitopes may differ between the two strains. Moreover, the deletion of *tarM*(*Se*) in E73 did not further reduce IgG binding. To exclude potential contributions of non-WTA antigens, we analyzed the binding of a previously described monoclonal IgG1 (mAb 4461) directed against RboP-WTA with α-GlcNAc (*38*). Notably, the E73 wild type bound mAb 4461 in a dose-dependent manner but much less effectively than RN4220. In contrast, E73 ∆*tarM*(*Se*) did not bind mAb 4461. Thus, TarM(Se) is essential for the binding of mAb 4461 to *S. epidermidis* E73 RboP-WTA but its glycosylation product may differ from that of TarM(Sa).

To further analyze whether TarM(Sa) and TarM(Se) differ in their activities, binding of pooled human IgG and mAb 4461 to E73 ∆*tarM*(*Se*) complemented with a plasmid-encoded copy of either *tarM*(*Sa*) or *tarM*(*Se*) was compared (Fig. 1B). Complementation with *tarM*(*Sa*) led to a strong and dose-dependent increase of IgG and mAb 4461 binding that exceeded by far the binding capacity of E73 wild type. In contrast, complementation with *tarM*(*Se*) only restored wild-type level binding of IgG and mAb 4461 but led to no further increase. These data indicate that TarM(Se) is functional and shapes the immunogenicity of *S. epidermidis* but that its glycosylation product may differ from that of TarM(Sa) in its capacity to bind human IgG and mAb 4461.

### *S. epidermidis* TarM(Se) incorporates glucose instead of GlcNAc into RboP-WTA

**Fig. 2.**
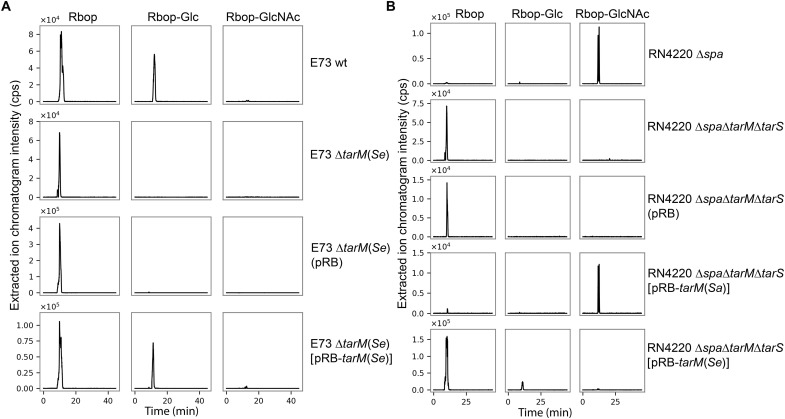
TarM(Se) glycosylates RboP-WTA with glucose rather than GlcNAc. (**A**) Mass spectroscopy–coupled high-performance liquid chromatography (HPLC-MS) demonstrates the *tarM*(*Se*)-dependent presence of RboP-glucose but the absence of RboP-GlcNAc in *S. epidermidis* E73 WTA. (**B**) Complementation of *S. aureus* RN4220 ∆*tarM*(*Sa*)∆*tarS* with *tarM*(*Sa*) restores the presence of RboP-GlcNAc but complementation with *tarM*(*Se*) allows the synthesis of RboP-glucose. Shown are extracted ion chromatograms.

While the composition of the E73 RboP-WTA backbone has recently been reported (*20*), the type of backbone glycosylation has not been analyzed yet. WTA isolated from E73 with or without *tarM*(*Se*) was analyzed by mass spectroscopy–coupled high-performance liquid chromatography (HPLC-MS) to detect sugar-modified RboP repeating units (Fig. 2). Notably, GlcNAc was absent from E73 RboP-WTA, while it could be detected in the WTA of *S. aureus* RN4220. Instead, the E73 wild type contained glucose-modified RboP repeating units, which were absent from those of RN4220. The deletion of *tarM*(*Se*) led to the absence of glucose but complementation with a *tarM*(*Se*) copy restored the wild-type phenotype, demonstrating that TarM(Se) is required for RboP modification with glucose. RboP units lacking glycosylation were also prominent in E73 wild type suggesting that TarM(Se) glycosylates only a subfraction of the RboP polymers or of the RboP repeating units of a given polymer (Fig. 2A). RN4220 lacking all WTA glycosyltransferases [∆*tarM*(*Sa*)*∆tarS*] complemented with *tarM*(*Se*) also lacked GlcNAc but contained glucose-modified RboP units (Fig. 2B).

To confirm that TarM(Sa) and TarM(Se) use different donor substrates, we set out to define the substrate specificity of TarM(Se). Four UDP-activated sugars, UDP-glucose, UDP-galactose, UDP–*N*-acetylgalactosamine, and UDP-GlcNAc, were used as donors for glycosylation. TarM(Se) was able to glycosylate purified poly(RboP) in a UDP-glucose–dependent manner, confirming that the enzyme has α-O-glucose transferase activity. However, TarM(Se) does not exclusively accept UDP-glucose as a donor substrate, it can also use UDP-galactose, although the latter is less efficient than UDP-glucose (table S1A). When purified poly(GroP) was used as an acceptor substrate, the activity of TarM(Se) was reduced to 2 to 30% compared with that for poly(RboP), indicating that TarM(Se) binds GroP-WTA less well (table S1B). Thus, *S. epidermidis* strains with *tarIJLM2* may use TarM(Se) with its altered glycosylation pattern to generate a WTA polymer that is less immunogenic and may support the bacteria in the evasion of host defense and, potentially, of phage infections.

### Phage Φ11 binds to glucose-modified RboP-WTA with similar efficacy as to GlcNAc-modified RboP-WTA

**Fig. 3.**
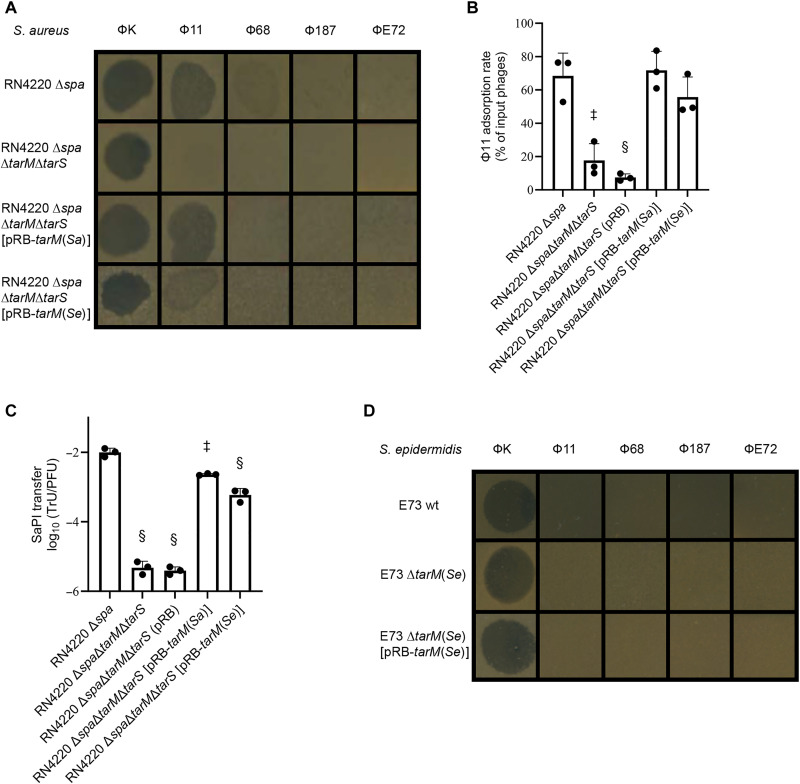
*S. aureus* phage Φ11 does not discriminate between RboP-WTA with GlcNAc or glucose. (**A**) RboP-WTA glycosylation with glucose does not affect the replication of Myovirus ΦK or Podovirus Φ68 in *S. aureus* RN4220 but allows the replication of Siphovirus Φ11 in a similar way as glycosylation with GlcNAc. (**B** and **C**) RboP-WTA glycosylation with glucose permits binding (B) and DNA transduction (C) by Φ11 in a similar way as glycosylation with GlcNAc. (**D**) Of the tested phages, *S. epidermidis* E73 only allows the replication of the broad host range Myovirus ΦK. Means ± SD of three independent experiments (three biological replicates) are shown. Significant differences versus RN4220∆*spa* (‡*P* < 0.001 and §*P* < 0.0001) were calculated by one-way ANOVA with Dunnett‘s posttest (two-sided) [(B) and (C)].

Glycosylated WTA represents the receptor structure for most of the known *Staphylococcus* phages, some of which can also discriminate between bacterial hosts with different glycosylation types (*28*, *34*, *39*). The currently known *S. epidermidis* phages only use GroP-WTA as a receptor and a *S. epidermidis* phage binding to RboP-WTA has never been found (*40*). To analyze how the replacement of GlcNAc by glucose on RboP-WTA may change the susceptibility to phages, the *S. aureus* RN4220 strain panel with or without *tarM*(*Sa*) or *tarM*(*Se*) was tested for susceptibility to a variety of *S. aureus*–specific phages (Fig. 3). Myovirus ΦK infected all strains, which confirms previous studies, which showed that ΦK requires only the WTA backbone for binding, irrespective of WTA glycosylation. Podovirus Φ68, which only infect *S. aureus* with RboP-WTA glycosylated by TarS with β-GlcNAc (*34*), and Siphoviruses Φ187 and ΦE72, which infect only the *S. aureus* lineage CC395 with GroP-WTA (*41*) or GroP-WTA producing *S. epidermidis* (*40*), respectively, did not infect any of the other strains. Siphovirus Φ11, however, which infects *S. aureus* strains with α-GlcNAc or β-GlcNAc glycosylation (*34*), also infected RN4220 with *tarM*(*Se*) as its only WTA glycosyltransferase gene (Fig. 3A). Likewise, *tarM*(*Se*) expression allowed Φ11 to bind to and to transduce RN4220 ∆*tarM*(*Sa*)*∆tarS* lacking its own WTA glycosyltransferases (Fig. 3, B and C). Thus, the receptor binding protein of Φ11 can accommodate either RboP-GlcNAc or RboP-glucose. None of the *S. aureus* phages except ΦK infected *S. epidermidis* E73, which was also resistant to the *S. epidermidis* phage ΦE72 (Fig. 3D).

### Three domains mediate different functions in TarM(Se)

**Fig. 4.**
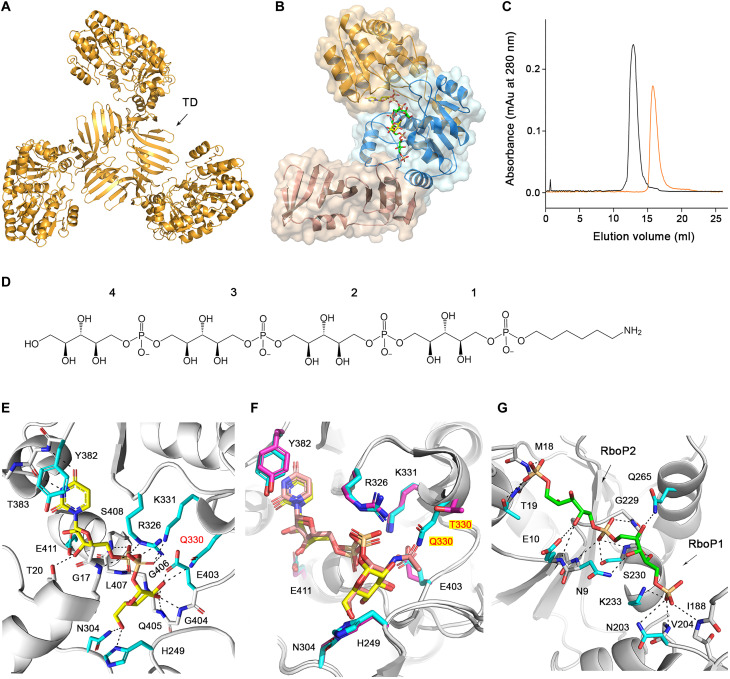
The overall structure of TarM(Se) and interactions of TarM(Se)_G117R_ with UDP-glucose or 4RboP-(CH_2_)_6_-NH_2_. (**A**) Crystal structure of TarM(Se) homotrimer. Trimerization domain (TD) is indicated. (**B**) Crystal structure of TarM(Se)_G117R_ monomer with product UDP (yellow) and 4RboP-glucose (4RboP, green; glucose at C4 position of the second unit of 4RboP, yellow). The nucleotide-binding domain (orange), acceptor-binding domain (blue), and TD (boron) are indicated. (**C**) Size exclusion chromatography elution profiles of TarM(Se) homotrimer (black) and TarM(Se)_G117R_ monomer (orange). On the basis of calibration of the column, TarM(Se) wild-type and TarM(Se)_G117R_ mutant proteins have estimated molecular weights of 162 kDa (*N* = 6) and 55 kDa (*N* = 8), respectively, in agreement with the calculated molecular weights of 180 kDa for a TarM(Se) homotrimer and 60 kDa for monomeric TarM(Se)_G117R_. mAu, milli-absorbance units. (**D**) Chemical structure of synthetic 4RboP-(CH_2_)_6_NH_2_. The unit numbers are indicated. (**E**) The binding site of UDP-glucose (yellow) in the TarM(Se)_G117R_–UDP-glucose complex structure with key amino acids (cyan), and Q330 was highlighted in red. Hydrogen bonds and salt bridges are shown as black dashed lines. (**F**) Superposition of TarM(Se)–UDP-glucose complex structure with TarM(Sa)-UDP-GlcNAc (PDB code 4X7M). The residues of TarM(Se) and TarM(Sa) are shown as cyan and magenta, respectively. UDP-glucose in TarM(Se) is colored yellow and UDP-GlcNAc in TarM(Sa) is salmon. The identical residues are labeled as black, and Q330 in TarM(Se) and T330 in TarM(Sa) are highlighted in red with a yellow background. (**G**) Interactions of TarM(Se)_G117R_ with 4RboP-(CH_2_)_6_NH_2_ (green) in the binary structure, and RboP1 and RboP2 are indicated.

To understand why the closely related TarM(Sa) and TarM(Se) proteins use different donor substrates, we solved the structure of unliganded full-length TarM(Se) from *S. epidermidis* at 3.2-Å resolution (Fig. 4A and table S2). Like its homolog TarM(Sa) (*32*, *33*), TarM(Se) forms a symmetric, propeller-like homotrimer, with three blades projecting from the central hub that mediates trimerization via its trimerization domain (TD; residues 69 to 201; Fig. 4, A and B) (*32*, *33*). Each blade of the homotrimer contains a catalytic domain with a canonical GT-B fold, consisting of an N-terminal acceptor substrate-binding domain (ABD; residues 1 to 68 and 202 to 302) and a C-terminal nucleotide-binding domain (residues 303 to 492; Fig. 4B). In line with this property, the elution profile of TarM(Se) from size exclusion chromatography corresponds to a molecular weight of 162 kDa (Fig. 4C), suggesting that it exists as homotrimer in solution.

Since the resolution of the native TarM(Se) structure was limited to 3.2 Å, we generated a G117R mutant [TarM(Se)_G117R_]. This mutation modifies an amino acid at the trimer interface and was designed to yield a monomeric protein that might form better-diffracting crystals (*33*). The mutant protein TarM(Se)_G117R_ formed crystals that diffracted to 2.06 Å (Table 1). TarM(Se)_G117R_ is monomeric both in the crystal (Fig. 4B) and in solution (Fig. 4C), and the activities of the native and mutant proteins are similar (table S3). To prepare complexes with reaction partners, a compound mimicking WTA, comprising four RboP repeating units [4RboP-(CH_2_)_6_NH_2_], was synthesized and used for cocrystallization (Fig. 4D and information S1 and S2). We obtained binary structures of TarM(Se)_G117R_ bound to either UDP-glucose or 4RboP-(CH_2_)_6_NH_2_. Furthermore, we solved the structure of a ternary complex of TarM(Se)_G117R_ bound to the product UDP and to glycosylated 4RboP-(CH_2_)_6_NH_2_. Analysis of the electron density in the ligand binding site clearly shows that the glycosylation reaction has taken place in the crystal.

**Table 1.** Data collection and refinement statistics for TarM(Se)_G117R_, TarM(Se)_G117R_–UDP-glucose, and TarM(Se)_G117R_-UDP-4RboP-glucose. Values in parentheses are for the highest-resolution shell. PDB, Protein Data Bank; RMS, root mean square.

	TarM(Se)_G117R_* (PDB code 7QD7)	TarM(Se)_G117R_–UDP-glucose* (PDB code 8P1X)	TarM(Se)_G117R_-UDP-4RboP-glucose* (PDB code 8P20)
**Data collection**			
Space group	P2_1_2_1_2_1_	P2_1_2_1_2_1_	P1
Cell dimensions			
*a*, *b*, *c* (Å)	58.68, 88.42, 97.49	58.65, 88.73, 98.05	58.62, 75.75, 129.40
α, β, γ (°)	90.00, 90.00, 90.00	90.00, 90.00, 90.00	90.01, 90.04, 90.03
Resolution (Å)	44.21–2.06 (2.11–2.06)	49.03–2.03 (2.08–2.03)	49.23–2.85 (2.92–2.85)
*R*_merge_	11.2 (165.6)	16.4 (198.4)	25.8 (181.9)
*I/*σ (*I*)	14.93 (1.55)	15.64 (1.42)	5.80 (0.88)
Completeness (%)	100.0 (100.0)	100.0 (100.0)	99.9 (100.0)
Redundancy	12.8 (12.5)	13.2 (13.1)	4.6 (4.2)
**Refinement**			
Resolution (Å)	43.71–2.06	49.03–2.03	49.21–2.85
No. of reflections	32047	33765	51972
*R*_work_/*R*_free_	21.85/23.61	20.10/23.81	22.65/26.32
No. of atoms			
Protein	3722	3847	14004
Ligand		36	356
Ions	9	18	3
Other molecules	35	33	64
Water	218	293	734
*B*-factors			
Protein	49.7	38.5	59.6
Ligand		38.4	60.7
Ions	52.1	49.0	75.5
Other molecules	54.6	48.1	72.4
Water	50.5	42.5	43.1
RMS deviations			
Bond lengths (Å)	0.004	0.004	0.003
Bond angles (°)	1.081	1.143	1.094

*Diffraction data from a single crystal were used to obtain the structure.

### Gln^330^ is a key residue for UDP-glucose binding

UDP-glucose is firmly held in a deep pocket through multiple contacts, leaving only the β-phosphate and glucose moiety exposed to the acceptor substrate (Fig. 4E, Table 1, and fig. S3A). The backbone amide and carbonyl groups of Thr^383^ form two hydrogen bonds with the O2 and N3 atoms of the base, providing specificity for uridine, and the aromatic ring system of the base is stacked against the Tyr^382^ side chain. The ribose moiety forms three interactions with the protein. The C2 and C3 hydroxyls interact with the Glu^411^ side chain, and the C3 hydroxyl is additionally hydrogen-bonded to the side chain of Thr^20^. The tandem backbone amide groups of Leu^407^ and Ser^408^ contact the α-phosphate of UDP-glucose and another main-chain amide group from Gly^17^ interacts with β-phosphate. In addition, the side chains of Arg^326^ and Lys^331^, each form two salt bridges with the β-phosphate. The glucose moiety contacts TarM(Se)_G117R_ through multiple interactions. The side chains of Asn^304^ and His^249^ are hydrogen-bonded to the C6 hydroxyl group, the backbone amide group of Gly^406^ interacts with C4 hydroxyl, and the Glu^403^ side chain and backbone amide groups of Gly^404^ and Gln^405^ contact with the C3 hydroxyl group. The side chain of Gln^330^ is hydrogen-bonded to the C2 hydroxyl. The related enzyme TarM(Sa), which accepts UDP-GlcNAc as donor substrate, has a threonine at this position (*32*). Thus, the longer Gln^330^ side chain appears to allow TarM(Se) to distinguish UDP-glucose from UDP-activated bulkier sugars, such as UDP-GalNAc and UDP-GlcNAc (table S1A). The binding site for UDP-glucose in TarM(Se) is composed of eight amino acids, seven of which are identical to that for UDP-GlcNAc in TarM(Sa) (*33*). The only difference is Gln^330^ in TarM(Se) and Thr^330^ in TarM(Sa), which clearly suggests a key role for this residue in allowing TarM(Se) to discriminate against the use of UDP-GlcNAc as a donor substrate (Fig. 4F). Analysis of all 13 HA-MRSE TarM(Se) sequences available in the BLAST database revealed that Gln^330^ is conserved in all copies of the gene, suggesting that all *S. epidermidis* strains with *tarIJLM2* can produce RboP-WTA carrying glucose.

### TarM(Se)_G117R_ binds poly(RboP) in the binary structure

The 4RboP-(CH_2_)_6_NH_2_ compound was introduced into the TarM(Se)_G117R_ crystals through cocrystallization. However, interpretable electron density was only observed for two RboP units and one phosphate group (Fig. 4G, fig. S3B, and table S2), suggesting that the remainder of the molecule is not ordered. We were not able to identify the unit number of 4RboP-(CH_2_)_6_NH_2_ due to the lack of electron density. After we obtained the ternary complex structure of TarM(Se)_G117R_ with product UDP and glycosylated 4RboP-(CH_2_)_6_NH_2_, the RboP unit number in the binding site could be assigned. The side chain of Lys^233^ forms a salt bridge with the phosphate group of RboP1 [the first RboP unit of 4RboP-(CH_2_)_6_NH_2_], and the Asn^203^ side chain, the backbone amide groups of Val^204^ and Ile^188^ contact the same phosphate. The ribitol moiety of RboP1 forms three hydrogen bonds with the side chains of Asn^9^ and Gln^265^, as well as the backbone carbonyl group of Gly^229^. Three main-chain amide groups interact with the phosphate group of RboP2. Two of these are from tandem backbone amide groups of Gly^229^ and Ser^230^, and the third one is contributed by Asn^9^; this phosphate group is also hydrogen-bonded to the side chain of Ser^230^. The ribitol moiety of RboP2 has only contact with the side chain and backbone amide group of Glu^10^. The phosphate group of RboP3 interacts with tandem backbone amide groups of Met^18^ and Thr^19^ and is further hydrogen-bonded to the Thr^19^ side chain. We could not find any electron density for the ribitol moiety of RboP3, the entire RboP4 unit, and the linker region of the molecule.

### Poly(RboP) is glycosylated by TarM(Se)_G117R_ in the crystal

**Fig. 5.**
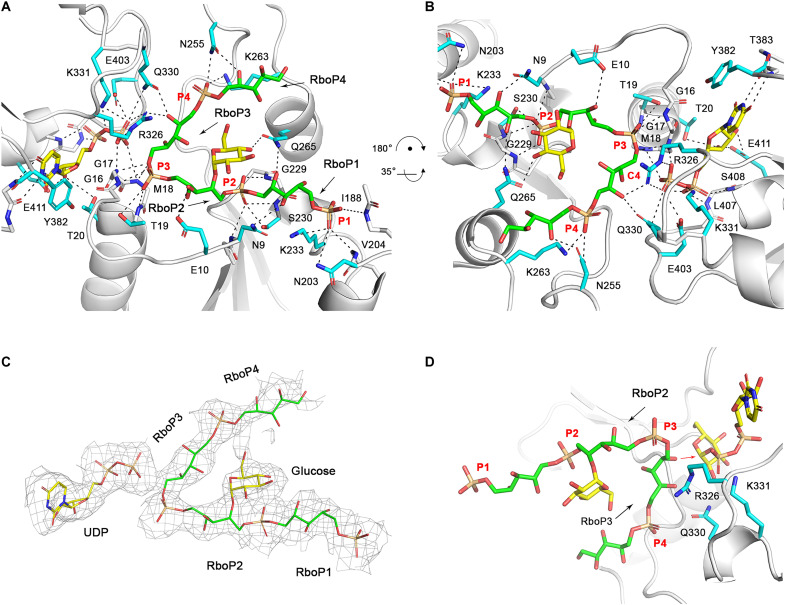
Interactions of TarM(Se)_G117R_ with product UDP and glycosylated 4RboP-(CH_2_)_6_NH_2_ (4RboP-glucose) and reaction mechanism of TarM(Se). (**A**) The binding sites of product UDP (yellow) and 4RboP-glucose in TarM(Se)_G117R_-UDP-4RboP-glucose complex structure with key amino acids (cyan), focusing on the binding site of 4RboP-glucose for clarity. The linker region is omitted because of no electron density. 4RboP is colored green and glucose on C4 of RboP2 is colored yellow. d-Ribitol 5-phosphate units, RboP1, RboP2, RboP3, and RboP4, are labeled. The binding sites for the phosphate group of RboP1, RboP2, RboP3, and RboP4 are indicated as P1, P2, P3, and P4, respectively. Hydrogen bonds and salt bridges are shown as black dashed lines. (**B**) Upon 180° and 35° rotation of (A), focusing on the active center. P1, P2, P3, and P4 for phosphate-binding sites and C4 hydroxyl of RboP3 in the active center are labeled. The unit numbers are omitted for clarity. (**C**) Simulated-annealing (mFo-DFc) omit map of UDP (yellow) and 4RboP-glucose in the TarM(Se)_G117R_-UDP-4RboP-glucose complex structure (gray mesh at 1.5σ). 4RboP is colored green and glucose residue on 4RboP is colored yellow. The product UDP, RboP unit numbers, and glucose residue on 4RboP are indicated. (**D**) View into the active center of TarM(Se). RboP2 and RboP3 are labeled. The red arrow indicates how the C4 hydroxyl of RboP3 could nucleophilically attack C1 of UDP-glucose on the α face.

**Fig. 6.**
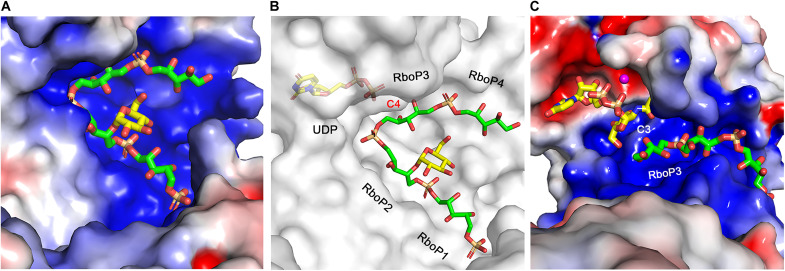
Electrostatic potential surface representation of 4RboP-glucose binding site in TarM(Se) and relative positions of donor and acceptor substrates in TarM(Se) and TarP from *S. aureus* (PDB code 6H4M). (**A**) Electrostatic potential surface representation of 4RboP-glucose binding site in TarM(Se), with electrostatic potential +5 kcal/mol in blue to −5 kcal/mol in red. 4RboP is colored green and glucose residue on 4RboP is colored yellow. (**B**) Relative position of product UDP (yellow) and 4RboP-glucose in TarM(Se)_G117R_, a retaining WTA glycosyltransferase, in surface presentation (gray). 4RboP-glucose is colored as in (A). The unit numbers and C4 hydroxyl of RboP3 in the active center are indicated. (**C**) Relative position of UDP-GlcNAc (yellow) and 3RboP (green) in TarP from *S. aureus*, an inverting WTA glycosyltransferase, in electrostatic potential surface representation, with +5 kcal/mol in blue to −5 kcal/mol in red. Mg^2+^ is shown as a ball, colored magenta. RboP3 and C3 hydroxyl of RboP3 in the active center are indicated.

Although crystals of TarM(Se)_G117R_ cocrystallized with 4RboP-(CH_2_)_6_NH_2_ were used for soaking of UDP-glucose and 4RboP-(CH_2_)_6_NH_2_, electron density was only observed for the product UDP and glycosylated 4RboP in the binding sites (Fig. 5C). This demonstrates that the glycosylation reaction has taken place, confirming that the crystallized protein is enzymatically active (*42*). Most of the interactions between UDP and TarM(Se)_G117R_ in the ternary complex are the same as those seen in the binary structure. For the binding site of 4RboP-(CH_2_)_6_NH_2_, the electron density for 4RboP is well defined and allows for unambiguous placement of the ligand, including its orientation. In the initial refinement, we noticed a disc-shaped, strong positive difference electron density that connected to C4 hydroxyl of RboP2 in all four copies of the TarM(Se)_G117R_ ternary complex in the asymmetric unit. We used 4RboP-glucose instead of 4RboP, did further refinement, and concluded that our ternary structure represents a complex of TarM(Se)_G117R_ with product UDP and glycosylated 4RboP that carries a glucose residue at the C4 position of RboP2 (4RboP-glucose) (Fig. 5 and Table 1). The interactions between RboP1 and TarM(Se)_G117R_ are all conserved as in the binary structure. Four interactions for the phosphate group of RboP2 in the binary structure are present in the ternary complex structure, while the ribitol moiety of RboP2 contacts the side chain of Glu^10^, and the glucose at C4 position is hydrogen-bonded to the Gln^265^ side chain. The phosphate group of RboP3 is fixed by two tandem backbone amide groups of Gly^16^, Gly^17^, Met^18^, and Thr^19^, and its ribitol moiety is hydrogen-bonded to the side chains of Arg^326^ and Gln^330^. The phosphate group of RboP4 forms a salt bridge with Lys^263^ and is further hydrogen-bonded to the side chain of Asn^255^, while the ribitol moiety also interacts with the Asn^255^ side chain (Fig. 5, A and B). For convenience of description, the binding sites for the phosphate groups of RboP1, RboP2, RboP3, and RboP4 are referred to as P1, P2, P3, and P4, respectively. As shown in Fig. 5, the key interactions between TarM(Se)_G117R_ and 4RboP-glucose are formed mainly by backbone amide groups that serve to anchor phosphate groups of 4RboP-glucose into the P1, P2, and P3 sites, while Arg^326^ and Gln^330^ side chains interact with the ribitol moiety of RboP3, with help from Lys^263^ and Asn^255^, leading the poly(RboP) fragment to adopt a V-shaped conformation, in which the phosphate group of RboP3 is located at the vertex. As a result of these interactions, 4RboP-glucose rests in an extended electropositive groove on the TarM(Se) surface (Fig. 6A). The observed binding mode for phosphate groups and the extended electropositive groove on the TarM(Se) surface is similar to that of 3RboP bound to TarP, a structure of an inverting WTA glycosyltransferase (Fig. 6C) that we have determined earlier (*28*). TarM(Se) is a retaining WTA glycosyltransferase; therefore, the relative positions of donor and acceptor substrates in TarM(Se) and TarP are different (Fig. 6, B and C).

### TarM(Se) catalyzes glycosylation reaction via an internal nucleophilic substitution (S_N_i)-like mechanism

As shown in Fig. 5, Arg^326^ and Lys^331^ both coordinate with the β-phosphate of the donor substrate, so that the glucose moiety lies in a correct orientation for the attacking of the nucleophile. Simultaneously, they could stabilize negative charges on the leaving group. The side chains of Arg^326^ and Gln^330^ interact not only with the donor substrate but also with 4RboP-glucose, with help from Lys^263^, enforcing the RboP unit in the active center to adopt a proper orientation for glycosylation. Our mutation analysis and previous studies show that the substitution of Arg^326^, Lys^331^, and Gln^330^ into alanine renders the enzyme inactive (table S3) (*32*, *33*). Thus, we propose that these three residues are essential for catalysis. Glu^403^ rests near the side chain of Lys^331^, the distances between the side chains of these two residues are similar in the TarM(Se)_G117R_–UDP-glucose binary structure and the ternary structure of TarM(Se)_G117R_-UDP-4RboP-glucose (2.76 and 3.03 Å, respectively), which indicates that the side chain of Glu^403^ could interact with Lys^331^, helping it in correct orientation during the catalytic cycle. In line with this assumption, the activities of E403A and K263A mutant proteins are severely reduced compared with that of the wild-type protein (table S3). Therefore, Glu^403^ and Lys^263^ are both important for binding and catalysis. Asn^9^, Glu^10^, Asn^203^, Lys^233^, Asn^255^, and Gln^265^ are involved in the binding of 4RboP-glucose, as alanine mutant proteins of these residues are all well folded and homotrimeric (fig. S3, C and D). K233A and E10A showed 21.7 and 30.9% remaining activities, respectively, while the other four mutant proteins displayed more than 50% activity, suggesting that a single mutation in this region is not sufficient to affect poly(RboP) binding due to the multiple interactions.

To interpret the catalytic mechanism of TarM(Se), we changed UDP in Fig. 5B into UDP-glucose and omitted most residues except Arg^326^, Gln^330^, and Lys^331^ (Fig. 5D). As Fig. 5D shows, the C4 hydroxyl of the unit RboP3 rests at the α face of UDP-glucose, the distance between the C4 hydroxyl of RboP3 and the putative anomeric C1 of UDP-glucose is 2.64 Å. Furthermore, at 3.34 Å, the β-phosphate O2B atom of UDP is well within the hydrogen bonding distance of the C4 hydroxyl of RboP3 (Fig. 5, B and C, and 6B). The observed geometry and distances nicely support an internal nucleophilic substitution (S_N_i)-like mechanism (*43*, *44*). In this mechanism, the phosphate group of UDP-glucose would serve as a base catalyst, activating the C4 hydroxyl of RboP3 and the activated nucleophile could attack the anomeric C1 of UDP-glucose on the α face, thus yielding an α-O-glycosylated RboP-WTA. The nucleoside diphosphate leaving group could be stabilized by the side chains of Arg^326^ and Lys^331^.

### TarM(Se) is a processive WTA glycosyltransferase

**Fig. 7.**
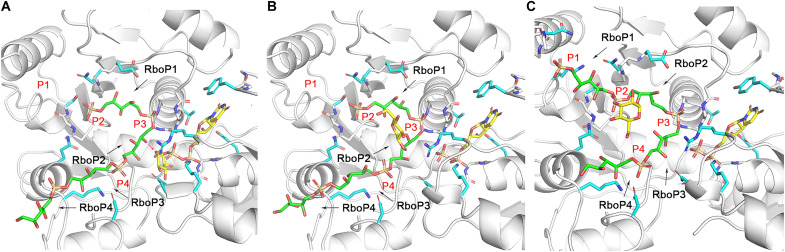
TarM(Se) is a processive WTA glycosyltransferase. (**A**) Binding of UDP-glucose (yellow) and 4RboP (green) in the proposed first step of the processive reaction [the linker region of 4RboP-(CH_2_)_6_NH_2_ is omitted for clarity]. The key amino acids in the ternary complex structure are shown (cyan). RboP1, RboP2, RboP3, and RboP4 are labeled. The binding sites of the phosphate group of RboP1, RboP2, RboP3, and RboP4 are labeled as P1, P2, P3, and P4, respectively, the same as in Fig. 2. The phosphate group of RboP2 is located at P3. The red arrow indicates that the C4 hydroxyl of RboP2 attacks nucleophilically C1 of UDP-glucose. (**B**) Glycosylation of 4RboP in the proposed second step of the processive reaction. Product UDP is colored yellow, 4RboP is colored green, and glucose residue on 4RboP is colored yellow. P1, P2, P3, and P4, and the unit numbers of 4RboP are indicated. (**C**) Crystal snapshot after sliding of 4RboP-glucose for one RboP unit. The phosphate group of RboP1 is shifted to the P1 site from P2. UDP and 4RboP-glucose are colored as in (B). P1, P2, P3, and P4, and unit numbers are labeled.

In *S. aureus*, all three enzymes, TarM(Sa), TarS, and TarP, glycosylate RboP-WTA with GlcNAc, but at the same or different positions in either α or β configuration (*25*, *27*, *28*). These glycosyltransferases are predicted to act as processive enzymes (*33*, *45*, *46*). However, so far, no structural evidence for this hypothesis is available. In our ternary complex structure, the UDP molecule occupies the binding site of UDP-glucose; the glucose residue is covalently bound at the C4 position of RboP2 in 4RboP-(CH_2_)_6_NH_2_, the phosphate group of RboP2 occupies P2 site, and the C4 hydroxyl of RboP3 is placed near to the β-phosphate of UDP (Figs. 5, 6B, and 7C), suggesting at least three reaction steps have been completed during the crystal soaking with UDP-glucose and 4RboP-(CH_2_)_6_NH_2_. First, UDP-glucose docks into its position, the RboP1 phosphate group of 4RboP-(CH_2_)_6_NH_2_ binds to the P2 site, and the phosphate groups of RboP2 and RBoP3 bind to P3 and P4 sites, respectively, putting the C4 hydroxyl of RboP2 at the active center for glycosylation (Fig. 7A). Second, glycosylation of the C4 hydroxyl of RboP2 occurs (Fig. 7B). Third, the glycosylated 4RboP-(CH_2_)_6_NH_2_ chain moves forward through the active center for one unit, so that the phosphate group of RboP1 is shifted to the P1 site just as the crystal snapshot of the ternary complex structure shows. We do not know how many steps really take place in the whole catalytic cycle of TarM(Se), but we think it is likely that the crystallographic snapshot of the active center in the ternary structure of TarM(Se)_G117R_-UDP-4RboP-glucose represents the rate-determining step of this cycle and this snapshot is just the step before the exchange of product UDP for UDP-glucose for the second catalytic cycle. Therefore, our ternary structure of TarM(Se)_G117R_ with UDP and 4RboP-glucose demonstrates (i) that TarM(Se) is a processive enzyme; (ii) that TarM(Se) starts the processive reaction from the second unit of the poly(RboP) chain, here RboP2; (iii) that the glycosylated poly(RboP) chain moves forward through the active center for one unit after each catalytic cycle; and (iv) that each RboP unit of the poly(RboP) chain except the first one is glycosylated, which is consistent with previous findings for *S aureus* RboP-WTA using nuclear magnetic resonance and MS analysis (*47*).

## DISCUSSION

**Fig. 8.**
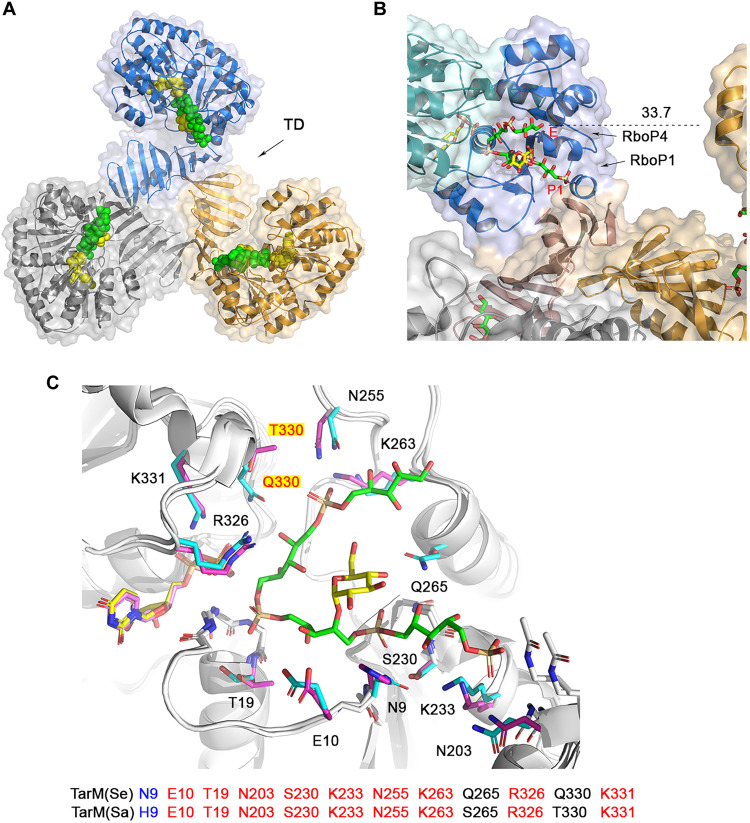
UDP and 4RboP-glucose in TarM(Se) homotrimer and comparison of the essential residues for catalysis and poly(RboP) binding in TarM(Se) with the corresponding residues in TarM(Sa) from *S. aureus* (PDB code 4X7R). (**A**) UDP and 4RboP-glucose in TarM(Se) homotrimer (blue, molecule A; orange, molecule B; gray, molecule C). UDP and 4RboP-glucose are shown as full-atom models. UDP is colored yellow, 4RboP is colored green, and glucose residue on 4RboP is colored yellow. TDs are indicated. (**B**) UDP and 4RboP-glucose in TarM(Se) homotrimer, focusing on molecule A. The nucleotide-binding domain (green), ABD (blue), and TD (boron) of molecule A are labeled. RboP1, RboP4, the entry site (E) of poly(RboP) chain into the active center, and the region of glycosylated poly(RboP) chain leaves TarM(Se) surface (near P1) are indicated. The distance (33.7 Å) from C1 hydroxyl of RboP4 in molecule A to the nearest point of molecule B is shown as a black dashed line. (**C**) Comparison of the essential residues for catalysis and poly(RboP) binding in TarM(Se) with the corresponding residues in TarM(Sa) from *S. aureus*. Residues of TarM(Se), UDP, and 4RboP-glucose are colored as in Fig. 2. TarM(Sa) residues are colored magenta and UDP is colored violet. Only residues of TarM(Se) and T330 in TarM(Sa) are labeled for clarity. Q330 in TarM(Se) and T330 in TarM(Sa) are highlighted in red with a yellow background. Key residues for catalysis and binding of 4RboP-glucose are shown at the bottom, with nine identical (red) and one conserved (blue).

We demonstrate here that TarM(Se) modifies RboP-WTA with glucose, in contrast to *S. aureus* TarM(Sa), which modifies RboP-WTA with GlcNAc. HA-MRSE clones with *tarIJLM2* may use the altered glycosylation pattern generated by TarM(Se) to support their immune evasion capacities. Our study also reveals phenotypic consequences of the replacement of GlcNAc with glucose on RboP-WTA. To decipher the reaction mechanism, we determined several structures of TarM(Se) in complex with ligands that together serve to outline the binding mode as well as the likely catalytic pathway of this glycosyltransferase. The ternary complex structure of TarM(Se)_G117R_ bound to UDP and 4RboP-glucose provides, for the first time, clear evidence that a glycosyltransferase glycosylates WTA in a processive manner. This is in line with the observations that WTAs extracted from bacterial cells are heavily modified with sugars (*23*, *47*), and the WTA chains contain sugars with exclusive α or β configuration (*48*, *49*). The enzyme activity and theoretical potential for the processive ability of the TarM(Sa) homotrimer and TarM(Sa)_G11R_ monomer are similar, suggesting that the trimerization does not affect the substrate binding, catalysis, and processivity (*33*). In our ternary complex structure, the ribitol moiety of RboP4 rests in the center of the ABD, while the phosphate group of RboP1 (P1 site) lies at the interface of ABD and TD, indicating that the poly(RboP) chain moves into the active center from the entry site (E), and the glycosylated poly(RboP) chain leaves the TarM(Se) surface near the P1 site (Fig. 8, A and B). The C1 hydroxyl group of RboP4 in molecule A is 33.7 Å away from molecule B in the homotrimer, which corresponds to a length of 3.7 RboP units. Therefore, it is not possible that three TarM(Se) molecules in the homotrimer glycosylate the same poly(RboP) chain at the same time in the processive reaction. This is consistent with the observation about similarities of the activity and processive ability of TarM(Sa) homotrimer and TarM(Sa)_G117R_ monomer (*33*). The processive ability of the trimeric TarS wild-type protein is 18-fold higher than that of a C-terminally truncated enzyme that lacks the TD, suggesting a contribution of the TD or trimerization to the processivity of TarS (*45*).

To date, no complex structure of TarM(Sa) with its donor and acceptor substrates is available. The ternary structure of TarM(Se)-UDP-4RboP-glucose allows us to predict also how poly(RboP) binds to the homologous TarM(Sa) enzyme (Fig. 8C). The binding sites for the four phosphate groups of 4RboP-glucose in TarM(Se) are all conserved in TarM(Sa), the essential residues for catalysis Arg^326^ and Lys^331^ are present in both enzymes, and the third one, Gln^330^, binds specifically the C2 hydroxyl of UDP-glucose in TarM(Se), while a threonine in TarM(Sa) accommodates the large volume of GlcNAc at C2 position (*33*). Of the other nine residues, which are in contact with 4RboP-glucose, eight are identical or conserved. Therefore, we conclude that the poly(RboP) chain adopts similar conformations in TarM(Sa) and in TarM(Se), and thus, the *S. aureus* TarM(Sa) is expected to use the same S_N_i-like mechanism for its catalytic reaction.

Glycosylated RboP-WTA has been considered a promising vaccine antigen against *S. aureus* because it is highly abundant on the bacterial surface and is largely invariant except for variation of the glycosidic linkages (*35*, *38*). Accordingly, the majority of anti–*S. aureus* antibodies in human sera are directed against glycosylated RboP-WTA (*35*). Our recent report of nosocomial *S. epidermidis* clones with RboP-WTA has raised hopes that such a vaccine could also protect from infections caused by *tarIJLM*-bearing *S. epidermidis* (*20*). Our study indicates that in addition to RboP-GlcNAc, a broadly active anti-*Staphylococcus* vaccine should also include RboP-glucose epitope to cover major invasive *S. epidermidis* clones such as ST10, ST23, and ST87. These *S. epidermidis* lineages do usually not colonize the human nose or arm skin but are often found as the cause of invasive infections (*20*). The reservoirs of nosocomial *S. epidermidis* clones, for instance, on skin parts that have not been analyzed for the presence of HA-MRSE clones yet, or in specific health care–associated habitats, have remained unknown. As *S. epidermidis* infections are usually restricted to hospitalized and immunocompromised patients, the adaptive immune systems of the majority of the human population have probably not been exposed to *tarIJLM*-expressing *S. epidermidis* clones, which may explain the apparent absence of IgG specific for RboP-glucose epitopes in pooled human sera. In addition, the type of glycosylation is crucial for the immunogenicity of RboP-WTA (*28*). While TarP and TarM(Sa), which are found only in a minority of the *S. aureus* strains and are dominant over the housekeeping RboP-WTA glycosyltransferase TarS, lead already to a substantial reduction in the immunogenicity of WTA (*28*, *38*), glycosylation by TarM(Se) may have the same or even a stronger impact on the immunogenic properties of RboP-WTA. In-depth immunological studies will unravel how the various glycosylation types affect the capacity of the human immune system to raise protective antibodies. Notably, coupling of TarP-modified RboP-WTA to an immunogenic carrier protein has been shown to restore full antigenicity of WTA (*50*), which opens attractive avenues for the development of anti-*Staphylococcus* vaccines directed against major WTA epitopes.

Nosocomial *S. epidermidis* clones have been shown to harness RboP-WTA because the additional polymer increases their persistence in the bloodstream (*20*). Future studies on the interaction of the various RboP-WTA glycosylation variants with human epithelial and endothelial receptors will reveal how WTA polymers with glucose contribute to the establishment of infection and evasion of immune responses. The replacement of GlcNAc with glucose on RboP-WTA did not alter the bacterial susceptibility of *S. aureus* to Siphovirus Φ11 and, potentially, other serogroup-B phages, which share similar receptor binding proteins. This finding extends our knowledge of the receptor and host preferences of *Staphylococcus*-specific phages and it will help in the development of phage therapy approaches, which will become promising alternatives for the treatment of multiresistant *S. aureus* and *S. epidermidis* infections (*51*). In addition, our ternary complex structure of TarM(Se)-UDP-4RboP-glucose, together with the structure of TarP-UDP-GlcNAc-3RboP (*28*), can now serve as a solid platform for the development of new inhibitors that could render MRSA and some of the abundant MRSE clones susceptible to human host defenses, and attenuate their virulence.

## MATERIALS AND METHODS

### Bacterial strains and growth conditions

*S. aureus* strain RN4220 was used for phage propagation and as a test strain for phage binding and transduction experiments. *S. aureus* JP1794 and PS187-H VW1 were used as donor strains for *Staphylococcus aureus* pathogenicity islands (SaPIs) particle propagation as described below. *Escherichia coli* DC10B was used as a cloning host. *E. coli* BL21(DE3) was used for protein expression. RN4220 and PS187 were used as donor strains for plasmid transduction. *S. epidermidis* and *S. aureus* strains were cultivated in tryptic soy broth (TSB) medium or Mueller-Hinton broth (MHB), or otherwise noted and incubated at 37°C on an orbital shaker. *E. coli* strains were cultivated in lysogeny broth (LB). Media were supplemented with appropriate antibiotics [tetracycline (5 μg/ml), chloramphenicol (10 μg/ml), or ampicillin (100 μg/ml)]. Clinical *S. epidermidis* strain E73 was from the strain collection used in our previously published study (*20*).

### Molecular genetic methods

For the construction of the ∆*tarM*(*Se*) mutant in *S. epidermidis* E73, the pBASE6-erm/lox1 shuttle vector was used according to standard procedures. For mutant complementation to *S. epidermidis* E73 and *S. aureus* RN4220, plasmid pRB474 was used. The primers for knockout and complementation plasmid construction are listed in table S4. The pRB474 with *tarM*(*Sa*) was constructed by the method and primers mentioned in a previous study. Plasmid transduction to *S. epidermidis* strains was performed using Φ11 with *S. aureus* RN4220 as donor strain or Φ187 with *S. aureus* PS187 as donor strain as described previously (*52*).

For TarM(Se) overexpression, the DNA sequence containing the coding region of *tarM*(*Se*) was chemically synthesized, inserted into pET-11α at NdeI and BamHI sites, and single mutations were introduced from the synthesized *tarM*(*Se*) using the same restriction sites (GenScript Biotech, Netherlands, B.V.). Obtained amplicons were confirmed by sequencing and were used to transform *E. coli* B21(DE3) for expression.

### IgG binding

*S. epidermidis* and *S. aureus* were grown overnight, washed, and adjusted to an OD_600_ (optical density at 600 nm) of 0.4 in phosphate-buffered saline (PBS) containing 0.1% bovine serum albumin. Twenty-five microliters of the diluted bacteria were incubated with 25-μl serial dilutions of pooled human IgG (Merck, I4506) or mAb 4461 In a 96-well plate for 30 min at 4°C (*38*, *53*). The samples were subsequently washed, centrifuged, and incubated with fluorescein isothiocyanate–labeled goat anti-human IgG F(ab′)2 FITC conjugate (2 μg/ml) (Merck, AQ112F) for 20 min at 4°C in the dark. Labeled bacteria were washed, centrifuged, and fixed with 1% paraformaldehyde for 20 min at room temperature in the dark. The bacteria were centrifuged again and resuspended in PBS, and surface-bound IgG was measured by flow cytometry using a BD FACSCalibur. Isotype control IgG (10 μg/ml) directed against HIV protein gp120 (b12-IgG) was used in experiments with mAb 4461. The whole bacterial population was gated, and the mean FL-1 fluorescence was analyzed with FlowJo version 10.8.1. The WTA-specific mAb 4461 and the B12 isotype control were described previously (*54*).

### Phage binding, infection, and transduction assays

Phage spot assays to test the bacterial susceptibilities were performed as described previously (*41*). Myovirus ΦK; Siphoviruses Φ11, Φ187, and ΦE72; and Podovirus Φ68 were freshly propagated in suitable bacterial host strains and filtered to yield sterile phage suspensions. Final concentrations of the phages were adjusted to approximately 1 × 10^9^ plaque-forming units (PFU)/ml. Test bacteria were cultivated overnight in fresh TSB to densities of OD_600_ = 0.5. One hundred microliters of the suspensions was added to 5 ml of soft agar for the preparation of bacterial overlay lawns. Ten microliters of each phage suspension was spotted on the bacterial lawns. After 37°C overnight incubation, phage-clearing zones were observed and recorded.

SaPI transfer experiments were performed according to standard procedures (*41*). Briefly, approximately 8.0 × 10^7^ cells of a recipient strain grown overnight were mixed with 100 μl of lysates obtained from *S. aureus* donor strain JP1794 or PS187-H VW1 bearing the tetracycline resistance marker-labeled SaPIbov1 ( ~1.0 × 10^6^ PFU ml^−1^) after the addition of 100 μl of transducing Φ11 lysate. Samples were then incubated for 15 min at 37°C, diluted, and plated on tetracycline-containing TSB agar to count transductant colonies.

The adsorption efficiency of ΦK, Φ11, Φ68, Φ187, and ΦE72 was determined as described previously with minor modifications (*41*). Briefly, adsorption rates were analyzed using a multiplicity of infection of 0.1. The adsorption rate was elucidated by determining the number of unbound phages in the supernatant and dividing the number of bound phages by the number of input phages.

### WTA isolation

Cell walls and WTA were isolated and purified according to previously described methods (*55*). Briefly, bacteria were grown in TSB (with 0.25% glucose) in a shaker at 37°C overnight. Bacterial cells were collected and disintegrated with a FastPrep-24 instrument (MP Biomedicals). The bacterial lysates were incubated with deoxyribonuclease I (Roche) and ribonuclease A (Sigma-Aldrich) at 37°C overnight. Cell walls were then obtained by sonification of lysates and repeated washing of the insoluble cell walls with 2% SDS solution. The WTA was released from peptidoglycan by treatment with 5% trichloroacetic acid, and then dialyzed in water using a 3.5 kDa molecular weight cut off (MWCO) Spectra/Por3 dialysis membrane (VWR International GmbH). Obtained soluble WTA was quantified by determining the content of phosphate, which corresponds to WTA amounts because each WTA repeating unit contains one phosphate residue as previously described (*55*). To quantify the WTA amount per cell, 300 μl of cell wall suspension was mixed with 300 μl of 1-M NaOH and incubated at 60°C with constant shaking of 600 rpm for 2 hours. The phosphate content in supernatants of this mixture was then measured by phosphate assay (*55*). The same amount of 300 μl of cell wall suspension was dried in a SpeedVac concentrator and weighed to determine the phosphate amount per cell wall dry mass.

### WTA compositional analysis

Identification of the WTA polymer type was performed using an Ultimate 3000RS HPLC system (Dionex) coupled to a micrOTOFII electrospray ionization (ESI)–time-of-flight (TOF) mass spectrometer (Bruker). Purified WTA was mixed 1:1 with 2-M NaOH and incubated at 60°C with constant shaking of 600 rpm for 2 hours, and then used in the composition analysis. For HPLC, a Gemini C18 column (150 mm × 4.6 mm, 110 Å, 5 μM, Phenomenex) was used at 37°C with a flow rate of 0.2 ml/min. A 5-min equilibration step with 100% buffer A (0.1% formic acid and 0.05% ammonium formate) was applied, followed by a linear gradient of 0 to 40% buffer B (acetonitrile) for 30 min. A final washing step with 40% buffer B for 5 min and a re-equilibration step (100% buffer A) for 5 min completed the method. Samples were ionized via ESI in positive ion mode. Exact masses in positive ion mode were presented as extracted ion chromatograms with DataAnalysis (Bruker). Base peak chromatograms were used for sample normalization.

### Semiquantitative biofilm assay

*S. epidermidis* biofilm formation was analyzed using 96-well delta microtiter plates (NUNC) as described previously (*20*) with the following modifications. Overnight cultures of bacterial cells were diluted in fresh TSB with 1% glucose and distributed into the 96-well plates with each well containing 200 μl of diluted bacterial cells. After incubation at 37°C for 1 hour, the bacterial cells in the plates were washed gently three times in PBS, and then stained with 0.1% crystal violet solution. The stain was washed off gently under slowly running water, and plates were dried. Last, 5% acetic acid was added to the wells to dissolve the staining. The absorbance was measured at 570 nm using a MicroELISA autoreader (Bio-Rad).

### Synthesis of 4RboP-(CH_2_)_6_NH_2_

4RboP-(CH_2_)_6_NH_2_ was synthesized according to the scheme described previously (information S1) (*56*). The analytic data can be found in information S2.

### Protein expression and purification

*E. coli* BL21(DE3) was grown in LB or TB medium at 30°C. Expression of *tarM*(*Se*) was induced with 0.5 mM isopropyl-β-d-thiogalactopyranoside at 22°C at an OD_600_ of 0.6. After 15 hours, cells were harvested, washed with wash buffer [50 mM Tris-HCl (pH 8.0) and 1 mM EDTA], and lysed by sonication in lysis buffer [70 mM NaH_2_PO_4_ (pH 8.0), 1 M NaCl, 10 mM β-mercaptoethanol, 20% glycerol, benzonase nuclease (10 U/ml)]. After centrifugation (15,000*g*), the supernatant was filtered with a 0.45-μm filter, loaded onto a HisTrap FF column (GE Healthcare, 5 ml), and washed with buffer A [50 mM NaH_2_PO_4_ (pH 8.0), 1 M NaCl, 10 mM β-mercaptoethanol, and 20% glycerol] supplemented with 42 mM imidazole and buffer B (buffer A with 60 mM imidazole). Last, the protein was eluted with buffer C (buffer A with 500 mM imidazole), and the fractions were pooled and further purified by size exclusion chromatography on a Superdex 200 increase 10/30 column equilibrated with buffer D {20 mM triethanolamine [pH 7.8] [for TarM(Se)_G117R_] or 8.5 [for TarM(Se) and mutant proteins], 250 mM LiCl, 10 mM β-mercaptoethanol, and 5% glycerol. The peak fractions were pooled and concentrated to 3.0 [for TarM(Se)] or 2.4 mg/ml [for TarM(Se)_G117R_] for crystallization.

### Glycosyltransferase activity assay

The activity of TarM(Se) and mutated proteins was determined with the ADP Quest Assay kit (DiscoverRx). The reaction volume was 20 μl with 1 mM UDP-glucose or other UDP-activated sugars, 1.5 mM purified poly(RboP) WTA from RN4220 Δ*tarM*(*Sa*)Δ*tarS* or poly(GroP) WTA from *S. epidermidis* E73 ∆*tarM*(*Se*). The reaction was started by the addition of proteins and incubated at room temperature for 1 hour. Released UDP was converted into a fluorescence signal that was detected in a 384-well black assay plate with 530-nm excitation and 590-nm emission wavelengths using TECAN Infinite M200.

### Circular dichroism

Circular dichroism measurements were performed on a JASCO J-720 spectropolarimeter (Gross-Umstadt, Germany). Purified TarM(Se) and mutant proteins (1.4 to 3.2 mg/ml in buffer D) were diluted with H_2_O to a final concentration of 0.2 mg/ml. A path length of 0.1 cm was used and the samples were scanned at a speed of 100 nm/min. Spectra were recorded at room temperature with an accumulation of 10 in the range of 250 to 190 nm and evaluated using the software Spectra Manager (Jasco).

### Crystallization and data collection

Crystals were obtained by hanging drop vapor diffusion. For native TarM(Se), 1 μl of protein solution (3 mg/ml) was mixed with 1 μl of reservoir solution containing 15% polyethylene glycol (PEG) 1000, 6 mM hexaamminecobalt(III) chloride, and 0.1 M tris-HCl (pH 6.9) at 12°C. For TarM(Se)_G117R_ mutant protein (2.4 mg/ml), the reservoir solution composed of 10% PEG 20,000, 25% PEG MME 550, 0.1 M MES/imidazole (pH 6.9), 0.03 M NaNO_3_, 0.03 M Na_2_HPO_4_, and 0.03 M (NH_4_)_2_SO_4_ at 20°C. The crystals of TarM(Se)_G117R_ were used for soaking UDP-glucose (26 mM) for 5 min. For crystals of TarM(Se)_G117R_ with 4RboP-(CH_2_)_6_NH_2_, 30 mM 4RboP-(CH_2_)_6_NH_2_ was introduced in the protein solution and 1 μl of protein solution was mixed with 1 μl of reservoir solution containing 10% PEG 20,000, 25% PEG MME 550, 0.1 M MES/imidazole (pH 6.9), 0.02 M sodium formate, 0.02 M ammonium acetate, 0.02 M trisodium citrate, 0.02 M sodium potassium tartrate (racemic), and 0.02 M sodium oxamate. The crystals of TarM(Se)_G117R_ with 4RboP-(CH_2_)_6_NH_2_ were used for soaking of UDP-glucose (20 mM) and 4RboP-(CH_2_)_6_NH_2_ (41 mM) together for 5 min.

For data collection, the crystals were cryo-protected with 20% 2-methyl-2,4-pentanediol in reservoir solution and flash-frozen in liquid nitrogen. Diffraction data were collected at beamline X06DA of Swiss Light Source in Villigen, Switzerland.

### Structure solution and refinement

All data were reduced using XDS/XSCALE software packages (*57*). The structure of native TarM(Se) was solved by molecular replacement using PHASER software, and a version of TarM(Sa) [Protein Data Bank (PDB) code 4WAC] was modified by CHAINSAW and then used as a search model (*32*, *58*, *59*). The final structure of native TarM(Se) was achieved by cycles of iterative model modification using COOT (*60*) and restrained refinement with BUSTER and REFMAC5 (*61*, *62*). One chain of TarM(Se) was then used as a search model to solve the structure of TarM(Se)_G117R_ by molecular replacement. The two binary structures and one ternary complex structure of TarM(Se)_G117R_ with UDP-glucose or 4RboP-(CH_2_)_6_NH_2_ or both together were solved by molecular replacement using PHASER and the unliganded TarM(Se)_G117R_ structure was used as a search model. UDP-glucose in TarM(Se)_G117R_–UDP-glucose structure, 4RboP-(CH_2_)_6_NH_2_ in TarM(Se)_G117R_-4RboP-(CH_2_)_6_NH_2_ structure, as well as UDP and 4RboP-glucose in the ternary complex structure were removed from the models to calculate the simulated annealing (mFo-DFc) omit maps using PHENIX (*63*). The coordinate and parameter files for 4RboP and 4RboP-glucose were calculated by the PRODRG server (*64*). All structure figures were generated by PyMOL and the models were evaluated using MolProbity (*65*, *66*). Statistics for the data collection and refinement are reported in Table 1 and table S2.

### Statistical information

Statistical analysis was performed using the Prism 8.0 package (GraphPad Software). *P* values of ≤ 0.05 were considered significant.

## Supplementary Materials

### This PDF file includes:

Figs. S1 to S3 Tables S1 to S4 Supplementary Information 1 and 2 References
