# Supplementary material for: Invasive Staphylococcus epidermidis uses a unique processive wall teichoic acid glycosyltransferase to evade immune recognition
Source: Sci Adv. 2023 Nov 24;9(47):eadj2641. doi: 10.1126/sciadv.adj2641 (PMC10672168; doi:10.1126/sciadv.adj2641)
Supplement: Supplementary file 1 — Figs. S1 to S3 Tables S1 to S4 Supplementary Information 1 and 2 References [file sciadv.adj2641_sm.pdf]

Science Advances
AAAS

Supplementary Materials for

Invasive *Staphylococcus epidermidis* uses a unique processive wall teichoic acid glycosyltransferase to evade immune recognition

Yinglan Guo *et al.*

Corresponding author: Thilo Stehle, thilo.stehle@uni-tuebingen.de;
Andreas Peschel, andreas.peschel@uni-tuebingen.de

Sci. Adv. **9**, eadj2641 (2023)
DOI: 10.1126/sciadv.adj2641

This PDF file includes:

Figs. S1 to S3
Tables S1 to S4
Supplementary Information 1 and 2
References

```
TarM(Se) MKQTYMIVNELDVNKGGMTTAMLTRSKFFLDNEISGDIITFDFKANYKDILKELVQSKKM 60
TarM(Sa) MKKIFMMVHELDVNKGGMTSSMFNRSKEFYDADIPADIVTFDYKGNYDEIIKALKKQGKM 60

TarM(Se) DKRTQMHNPFIYFKNISNLQHKKYNYTMTRNLSNLLKDSVEIKENSRISRFFNIMSGEYL 120
TarM(Sa) DRRTKMYNVFEYFKQISNNKHFKSNKLLYKHISERLKNTIEIEESKGISRYFDITTGTYI 120

TarM(Se) AYKRETEQETIFDLFKNNLRYKRIYFYKGKIVKTEVFNSDNNLIAEQFYDDNGYLYLYRQ 180
TarM(Sa) AYIRKSKSEKVIDFFKDNKRIERFSFIDNKVHMKETFNVDNKVCYQVFYDEKGYPYISRN 180

TarM(Se) INPEKKSIGKTYLVCKEKQFKNNVEFCSYFLDKLIPDINDNIIICDGPGSFPKILKTNHK 240
TarM(Sa) INANNGAVGKTYVLVNKKEFKNNLALCVYYLEKLIKDSKDSIMICDGPGSFPKMFNTNHK 240

TarM(Se) NVKKFAVIHVNHYKNFDDTGAVKKQEDYILRNANKINGVVMLTEAQKKDIIEKYKITNAY 300
TarM(Sa) NAQKYGVIHVNHHENFDDTGAFKKSEKYIIENANKINGVIVLTEAQRLDILNQFDVENIF 300

TarM(Se) VISNFINITDDYRDKNDNKVVGHISRLVPQKGLPYLIDVAKKVVEQDNSVEFHLYGTGEE 360
TarM(Sa) TISNFVKIHNAPKHFQTEKIVGHISRMVPTKRIDLLIEVAELVVKKDNAVKFHIYGEGSV 360

TarM(Se) KSKIENLIQESNLTNNVKLLGYTTNAIEKIKDFRCVISTSQFEGQGLSLIEAMLLKKPVV 420
TarM(Sa) KDKIAKMIEDKNLERNVFLKGYTTTPQKCLEDFKLVVSTSQYEGQGLSMIEAMISKRPVV 420

TarM(Se) AFDVKYGPSDFVKDGKNGYLIENKDIKKMANKILKLLHDKELSKSLGKHGRDTIIDMYQP 480
TarM(Sa) AFDIKYGPSDFIEDNKNGYLIENHNINDMADKILQLVNNDVLAAEFGSKARENIIEKYST 480

TarM(Se) EKLMVKWKQLFN- 492
TarM     ESILEKWLNLFNS 493
```

Fig. S1. Sequence alignment of TarM(Se) from *S. epidermidis* and TarM(Sa) from *S. aureus*. The alignment was calculated with pairwise sequence alignment tool GGSEARCH2SEQ (*67*). Identical amino acids are shown in black boxes and similar amino acids are shaded in gray. The amino acids that are involved in binding of UDP-glucose (red), 4RboP-glucose (green) and catalysis (magenta) are indicated as filled triangle.

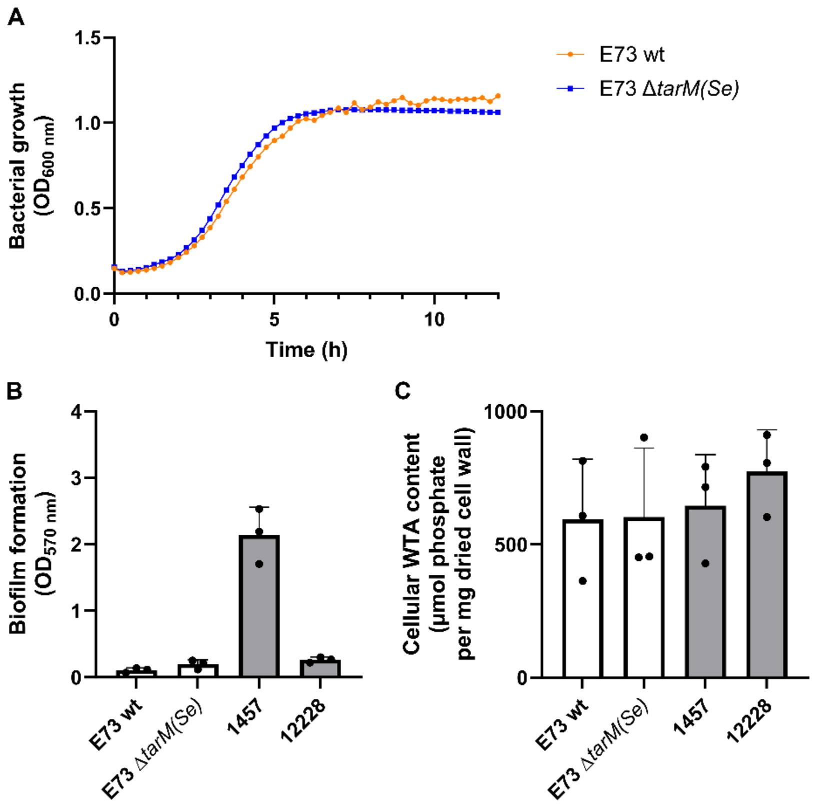

Fig. S2. TarM(Se) expression does not change the overall WTA amounts and does not affect *S. epidermidis* biofilm formation or growth behavior. (**A**) Growth curves of *S. epidermidis* E73 with or without *tarM(Se)* in TSB. Means of three independent experiments are shown. (**B**) The characteristic of negative biofilm formation in E73 is not altered by deletion of *tarM(Se*). The laboratory *S. epidermidis* strains ATCC12228 (biofilm negative) and 1457 (biofilm positive) were included as control strains. (**C**) Total WTA phosphate amount per cell wall dry weight of the indicated *S. epidermidis* strains. None of the minor differences is significant. Means ± s.d. of three independent experiments are shown in (**B**) and (**C**).

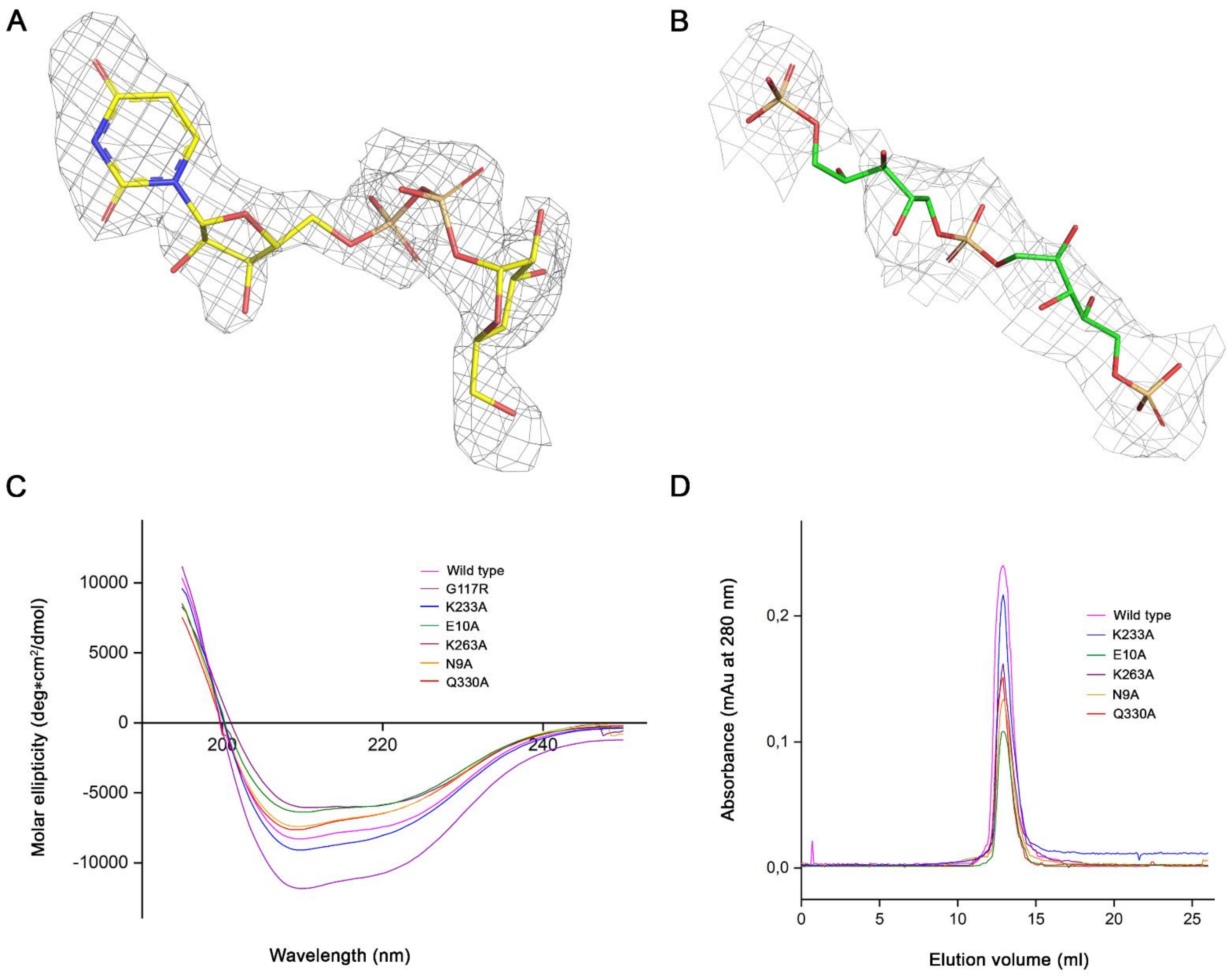

Fig. S3. Simulated-annealing (mFo − DFc) omit maps of UDP-glucose and 4RboP-$(CH_2)_6NH_2$ in the binary structures and characterization of TarM(Se) wild type and mutant proteins. (**A**) Simulated-annealing (mFo − DFc) omit map of UDP-glucose (yellow) in $\text{TarM(Se)}_{\text{G117R}}$-UDP-glucose complex structure (grey mesh, at 2.0 σ). (**B**) Simulated-annealing (mFo − DFc) omit map of 4RboP-$(CH_2)_6NH_2$ (green) in $\text{TarM(Se)}_{\text{G117R}}$-4RboP-$(CH_2)_6NH_2$ complex structure (grey mesh, at 1.5 σ). (**C**) Circular dichroism spectra of wild type and mutant TarM(Se) proteins (for wild type, G117R, Q330A and K263A, N = 3; for N9A, E10A, and K233A, N = 2). (**D**) Size exclusion chromatography elution profiles of wild-type and mutant TarM(Se) proteins (for wild type N = 6; for Q330A, K263A and E10A, N = 3; for N9A and K233A, N = 2).

Table S1. Substrate specificity of TarM(Se).

(**A**) Donor substrate specificity of TarM(Se) using poly(RboP) WTA as acceptor substrate.

Sugar nucleotide	Enzymatic activity (nmol/mg*min)	Activity of UDP-glucose in %
UDP-glucose	1.85	100
UDP-galactose	0.98	53
UDP-GalNAc	0.53	29
UDP-GlcNAc	0.3	16

(**B**) Donor substrate specificity of TarM(Se) using poly(GroP) WTA as acceptor substrate.

Sugar nucleotide	Enzymatic activity (nmol/mg*min)	Activity of UDP-glucose and poly(RboP) WTA in %
UDP-glucose	0.55	30
UDP-galactose	0.46	25
UDP-GalNAc	0.05	3
UDP-GlcNAc	0.04	2

Table S2. Data collection and refinement statistics for TarM(Se) and TarM(Se)$_{G117R}$-4RboP-(CH_2)$_6$$NH_2$.

	TarM(Se) native* (PDB 7QNT)	TarM(Se)$_{G117R}$-4RboP-(CH_2)$_6$$NH_2$*(PDB 7QH9)
**Data collection**		
Space group	$P6_3$	P1
Cell dimensions		
a, *b*, *c* (Å)	154.01, 154.01, 207.72	58.87, 70.57, 137.38
α, β, γ (°)	90.00, 90.00, 120.00	89.97, 90.01, 90.02
Resolution (Å)	48.99-3.21 (3.29-3.21)	49.23-2.69 (2.76-2.69)
R_{merge}	20.9 (229.8)	6.1 (142.7)
$I / \sigma(I)$	15.57 (1.64)	9.98 (0.70)
Completeness (%)	100.0 (100.0)	95.2 (97.4)
Redundancy	20.6 (20.7)	2.5 (2.5)
**Refinement**		
Resolution (Å)	48.99-3.21	49.21-2.69
No. reflections	45535	58472
R_{work} / R_{free}	22.77/25.72	24.26/25.28
No. atoms		
Protein	13389	14001
Ligand		124
Ions	16	3
other molecules	12	4
Water	264	240
B-factors		
Protein	93.7	97.9
Ligand		99.7
Ions	77.4	38.4
other molecules	91.2	29.4
Water	52.8	42.5
R.m.s. deviations		
Bond lengths (Å)	0.002	0.004
Bond angles (°)	1.020	1.296

Values in parentheses are for the highest-resolution shell. *Diffraction data from a single crystal were used to obtain the structure.

Table S3. Enzymatic activities of mutated TarM(Se) proteins.

Function	TarM(Se) variant	Activity of wild type in %
Trimer interface	G117R	96.0
Catalysis	R326A	Not active (*32*, *33*)
	Q330A	5.7
	K331A	Not active (*32*, *33*)
	E403A	Not active (*32*, *33*)
Poly(RboP) binding	N9A	51.0
	E10A	30.9
	N203A	64.7
	K233A	21.7
	N255A	100.1
	K263A	17.2
	Q265A	67.6

Table S4. Primers used in this study.

Primer name	primer sequence
***tarM(Se)* knockout**	
tarM(Se) pBASE 1up	CGATGGTACCGCTTTATTTAAAAGAAATATATCTGATAGAAG
tarM(Se) pBASE 1dn	TAATATTACCTCATTATTTATTTCTTAAATGC
tarM(Se) pBASE 2up	TAAATAATGAGGTAATATTAGTATCCATTTTTCTATTATTCAGTTTCTATGTAC
tarM(Se) pBASE 2dn	GTCAGTCGACCTATTTGACTATTATCAACTTTCTTCGCTTTATG
***tarM(Se)* expression in pRB474**	
*tarM(Se)*_F	GTCGGATCCAAAGGAGGTTATATA ATGAAACAAACTTATATGATTGTAAATGAGTTGG
*tarM(Se)*_R	CCGATGAATTCTTAGTTAAACAATTGTTTCCATTTCACCATC

Supplementary Information 1

4RboP-$(CH_2)_6NH_2$ was synthesized according to the following scheme as described previously (*56*).

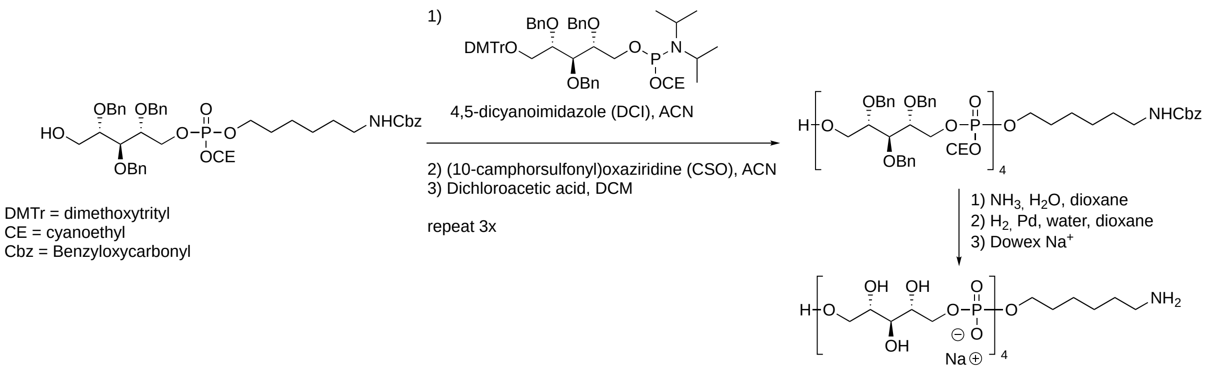

Supplementary Information 2

Analytical data:

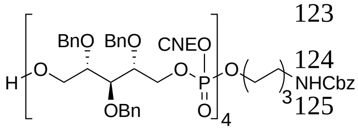

IR (neat, cm^{-1}): 3447, 2938, 2866, 1717, 1506, 1456, 1267, 1028, 1009, 746, 698; ^{1}H NMR (400 MHz, CD_3CN) δ = 1.27 (m, 4H, CH_2-hexyl spacer), 1.40 - 1.41 (m, 2H, CH_2-hexyl spacer), 1.58 - 1.59 (m, 2H, CH_2-hexyl spacer), 2.52 - 2.59 (m, 8H, 4x CH_2-cyanoethyl), 3.06 (q, 2H, J= 6.4 Hz, CH_2-N hexyl spacer), 3.67 - 3.79 (m, 3H, CH-Rbo, CH_2-Rbo), 3.84 - 4.13 (m, 22H, 12x CH-Rbo, CH_2-O hexyl spacer, 4x CH_2 cyanoethyl), 4.17 - 4.40 (m, 14H, 7x CH_2-Rbo), 4.50 - 4.69 (m, 24H, 12x CH_2-Bn), 5.05 (s, 2H, CH_2-Cbz), 5.72 (bs, 1H, N-H), 7.25 - 7.35 (m, 65H, H-arom); ^{13}C-APT NMR (101 MHz, CD_3CN) δ = 20.1, 20.2, 20.2 (CH_2 cyanoethyl), 25.7, 26.8, 30.4, 30.7, 30.8 (CH_2 hexyl spacer), 41.4 (CH_2-N hexyl spacer), 61.5 (CH_2-Rbo), 63.1, 63.1, 63.2 (CH_2 cyanoethyl), 66.6 (CH_2-Cbz), 67.5, 67.7, 67.8, 68.3 (CH_2-Rbo), 68.9, 69.0 (CH_2-O hexyl spacer), 72.7, 73.0, 73.1, 73.1, 74.5, 74.5, 74.6 (CH_2-Bn), 78.3, 78.6, 78.9, 78.9, 79.0, 79.1, 80.6 (CH-Rbo), 118.3 - 118.6 (C_q-cyanoethyl), 128.4, 128.6, 128.6, 128.7, 128.8, 128.9, 128.9, 129.3, 129.3, 129.4 (CH-arom), 139.1, 139.2, 139.3, 139.5, 139.7 (C_q-arom), 158.0 (C=O); ^{31}P NMR (162 MHz, CD_3CN) δ = 0.2, 0.2, 0.2, -0.0, -0.1, -0.1, -0.2, -0.2; HRMS: $[M+H]^+$ calculated for $C_{101}H_{119}N_4O_{24}P_3$ 2401.9343, found 2401.9241.

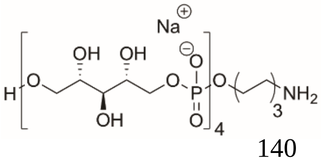

^{1}H NMR (600 MHz, D_2O) δ = 1.39 - 1.40 (m, 4H, 2x CH_2 hexyl spacer), 1.55 - 1.68 (m, 4H, 2x CH_2-hexyl spacer), 2.97 (t, 2H, J= 7.2 Hz, CH_2-N hexyl spacer), 3.62 (dd, 1H, J= 12.0 Hz, J= 7.2 Hz, CH_2-ribitol), 3.72 (t, 1H, J= 6.6 Hz, CH-ribitol), 3.76 - 3.82 (m, 4H, CH/CH_2-ribitol), 3.82 - 3.84 (m, 1H, CH/CH_2-ribitol), 3.85 - 3.96 (m, 16H, 14 CH/CH_2-ribitol, CH_2-O hexyl spacer), 3.97 - 4.06 (m, 7H, CH/CH_2-ribitol); ^{13}C-APT NMR (151 MHz, D_2O) δ = 25.4, 26.0, 27.5 (3x CH_2-hexyl spacer), 30.3 (d, J= 7.6 Hz, CH_2-hexyl spacer), 40.3 (CH_2-N hexyl spacer), 63.2 (CH_2-ribitol), 67.0 - 67.4 (7x CH_2-ribitol/CH_2-O hexyl spacer), 71.7 - 73.0 (10x CH-ribitol); ^{31}P NMR (162 MHz, D_2O) δ = 1.8, 1.6; HRMS: $[M+H]^+$ calculated for $C_{26}H_{60}NO_{29}P_4$ 974.2201, found 974.2202.

REFERENCES AND NOTES

1. M. Otto, *Staphylococcus epidermidis*—The 'accidental' pathogen. *Nat. Rev. Microbiol.* **7**, 555–567 (2009).

2. M. M. Severn, A. R. Horswill, *Staphylococcus epidermidis* and its dual lifestyle in skin health and infection. *Nat. Rev. Microbiol.* **21**, 97–111 (2023).

3. A. L. Byrd, Y. Belkaid, J. A. Segre, The human skin microbiome. *Nat. Rev. Microbiol.* **16**, 143–155 (2018).

4. A. Kengmo Tchoupa, D. Kretschmer, B. Schittek, A. Peschel, The epidermal lipid barrier in microbiome-skin interaction. *Trends Microbiol.* **31**, 723–734 (2023).

5. M. Li, X. Wang, Q. Gao, Y. Lu, Molecular characterization of *Staphylococcus epidermidis* strains isolated from a teaching hospital in Shanghai, China, *J. Med. Microbiol.* **58**, 456–461 (2009).

6. E. A. Grice, J. A. Segre, The skin microbiome. *Nat. Rev. Microbiol.* **9**, 244–253 (2011).

7. K. Becker, C. Heilmann, G. Peters, Coagulase-negative staphylococci. *Clin. Microbiol. Rev.* **27**, 870–926 (2014).

8. S. Naik, N. Bouladoux, C. Wilhelm, M. J. Molloy, R. Salcedo, W. Kastenmuller, C. Deming, M. Quinones, L. Koo, S. Conlan, S. Spencer, J. A. Hall, A. Dzutsev, H. Kong, D. J. Campbell, G. Trinchieri, J. A. Segre, Y. Belkaid, Compartmentalized control of skin immunity by resident commensals. *Science* **337**, 1115–1119 (2012).

9. G. J. Christensen, H. Bruggemann, Bacterial skin commensals and their role as host guardians. *Benef. Microbes* **5**, 201–215 (2014).

10. Q. Liu, Q. Liu, H. Meng, H. Lv, Y. Liu, J. Liu, H. Wang, L. He, J. Qin, Y. Wang, Y. Dai, M. Otto, M. Li, *Staphylococcus epidermidis* contributes to healthy maturation of the nasal microbiome by stimulating antimicrobial peptide production. *Cell Host Microbe* **27**, 68–78.e5 (2020).

11. T. Iwase, Y. Uehara, H. Shinji, A. Tajima, H. Seo, K. Takada, T. Agata, Y. Mizunoe, *Staphylococcus epidermidis* Esp inhibits *Staphylococcus aureus* biofilm formation and nasal colonization. *Nature* **465**, 346–349 (2010).

12. S. Y. Tong, J. S. Davis, E. Eichenberger, T. L. Holland, V. G. Fowler, Jr., *Staphylococcus aureus* infections: Epidemiology, pathophysiology, clinical manifestations, and management. *Clin. Microbiol. Rev.* **28**, 603–661 (2015).

13. A. S. Lee, H. de Lencastre, J. Garau, J. Kluytmans, S. Malhotra-Kumar, A. Peschel, S. Harbarth, Methicillin-resistant *Staphylococcus aureus*. *Nat. Rev. Dis. Primers.* **4**, 18033 (2018).

14. W. Ziebuhr, S. Hennig, M. Eckart, H. Kränzler, C. Batzilla, S. Kozitskaya, Nosocomial infections by *Staphylococcus epidermidis*: How a commensal bacterium turns into a pathogen. *Int. J. Antimicrob. Agents* **28**, S14–20 (2006).

15. G. Méric, L. Mageiros, J. Pensar, M. Laabei, K. Yahara, B. Pascoe, N. Kittiwan, P. Tadee, V. Post, S. Lamble, R. Bowden, J. E. Bray, M. Morgenstern, K. A. Jolley, M. C. J. Maiden, E. J. Feil, X. Didelot, M. Miragaia, H. de Lencastre, T. Fintan Moriarty, H. Rohde, R. Massey, D. Mack, J. Corander, S. K. Sheppard, Disease-associated genotypes of the commensal skin bacterium *Staphylococcus epidermidis*. *Nat. Commun.* **9**, 5034 (2018).

16. M. Otto, Molecular basis of *Staphylococcus epidermidis* infections. *Semin. Immunopathol.* **34**, 201–214 (2012).

17. X. Du, Y. Zhu, Y. Song, T. Li, T. Luo, G. Sun, C. Yang, C. Cao, Y. Lu, M. Li, Molecular analysis of *Staphylococcus epidermidis* strains isolated from community and hospital environments in China. *PLOS ONE* **8**, e62742 (2013).

18. J. Y. H. Lee, I. R. Monk, A. Gonçalves da Silva, T. Seemann, K. Y. L. Chua, A. Kearns, R. Hill, N. Woodford, M. D. Bartels, B. Strommenger, F. Laurent, M. Dodémont, A. Deplano, R. Patel, A. R. Larsen, T. M. Korman, T. P. Stinear, B. P. Howden, Global spread of three multidrug-resistant lineages of *Staphylococcus epidermidis*. *Nat. Microbiol.* **3**, 1175–1185 (2018).

19. K. Y. Le, M. D. Park, M. Otto, Immune evasion mechanisms of *Staphylococcus epidermidis* biofilm infection. *Front. Microbiol.* **9**, 359 (2018).

20. X. Du, J. Larsen, M. Li, A. Walter, C. Slavetinsky, A. Both, P. M. Sanchez Carballo, M. Stegger, E. Lehmann, Y. Liu, J. Liu, J. Slavetinsky, K. A. Duda, B. Krismer, S. Heilbronner, C. Weidenmaier, C. Mayer, H. Rohde, V. Winstel, A. Peschel, *Staphylococcus epidermidis* clones express *Staphylococcus aureus*-type wall teichoic acid to shift from a commensal to pathogen lifestyle. *Nat. Microbiol.* **6**, 757–768 (2021).

21. C. Weidenmaier, A. Peschel, Teichoic acids and related cell-wall glycopolymers in Gram-positive physiology and host interactions. *Nat. Rev. Microbiol.* **6**, 276–287 (2008).

22. A. Oren, G. M. Garrity, Valid publication of the names of forty-two phyla of prokaryotes. *Int. J. Syst. Evol. Microbiol.* **71**, 005056 (2021).

23. S. Brown, J. P. Santa Maria Jr., S. Walker, Wall teichoic acids of gram-positive bacteria. *Annu. Rev. Microbiol.* **67**, 313–336 (2013).

24. R. van Dalen, A. Peschel, N. M. van Sorge, Wall teichoic acid in *Staphylococcus aureus* host interaction. *Trends Microbiol.* **28**, 985–998 (2020).

25. S. Brown, G. Xia, L. G. Luhachack, J. Campbell, T. C. Meredith, C. Chen, V. Winstel, C. Gekeler, J. E. Irazoqui, A. Peschel, S. Walker, Methicillin resistance in *Staphylococcus aureus* requires glycosylated wall teichoic acids. *Proc. Natl. Acad. Sci. U.S.A.* **109**, 18909–18914 (2012).

26. Y. Guo, N. M. Pfahler, S. L. Volpel, T. Stehle, Cell wall glycosylation in *Staphylococcus aureus*: Targeting the tar glycosyltransferases. *Curr. Opin. Struct. Biol.* **68**, 166–174 (2021).

27. G. Xia, L. Maier, P. Sanchez-Carballo, M. Li, M. Otto, O. Holst, A. Peschel, Glycosylation of wall teichoic acid in *Staphylococcus aureus* by TarM. *J. Biol. Chem.* **285**, 13405–13415 (2010).

28. D. Gerlach, Y. Guo, C. de Castro, S. H. Kim, K. Schlatterer, F. F. Xu, C. Pereira, P. H. Seeberger, S. Ali, J. Codée, W. Sirisarn, B. Schulte, C. Wolz, J. Larsen, A. Molinaro, B. L. Lee, G. Xia, T. Stehle, A.

Peschel, Methicillin-resistant *Staphylococcus aureus* alters cell wall glycosylation to evade immunity. *Nature* **563**, 705–709 (2018).

29. V. Winstel, P. Kühner, F. Salomon, J. Larsen, R. Skov, W. Hoffmann, A. Peschel, C. Weidenmaier, Wall teichoic acid glycosylation governs *Staphylococcus aureus* nasal colonization. *mBio* **6**, e00632 (2015).

30. M. Otto, Coagulase-negative staphylococci as reservoirs of genes facilitating MRSA infection: Staphylococcal commensal species such as *Staphylococcus epidermidis* are being recognized as important sources of genes promoting MRSA colonization and virulence. *Bioessays* **35**, 4–11 (2013).

31. G. Méric, M. Miragaia, M. de Been, K. Yahara, B. Pascoe, L. Mageiros, J. Mikhail, L. G. Harris, T. S. Wilkinson, J. Rolo, S. Lamble, J. E. Bray, K. A. Jolley, W. P. Hanage, R. Bowden, M. C. J. Maiden, D. Mack, H. de Lencastre, E. J. Feil, J. Corander, S. K. Sheppard, Ecological overlap and horizontal gene transfer in *Staphylococcus aureus* and *Staphylococcus epidermidis*. *Genome Biol. Evol.* **7**, 1313–1328 (2015).

32. C. Koç, D. Gerlach, S. Beck, A. Peschel, G. Xia, T. Stehle, Structural and enzymatic analysis of TarM glycosyltransferase from *Staphylococcus aureus* reveals an oligomeric protein specific for the glycosylation of wall teichoic acid. *J. Biol. Chem.* **290**, 9874–9885 (2015).

33. S. Sobhanifar, L. J. Worrall, R. J. Gruninger, G. A. Wasney, M. Blaukopf, L. Baumann, E. Lameignere, M. Solomonson, E. D. Brown, S. G. Withers, N. C. Strynadka, Structure and mechanism of *Staphylococcus aureus* TarM, the wall teichoic acid α-glycosyltransferase. *Proc. Natl. Acad. Sci. U.S.A.* **112**, E576–585 (2015).

34. X. Li, D. Gerlach, X. du, J. Larsen, M. Stegger, P. Kühner, A. Peschel, G. Xia, V. Winstel, An accessory wall teichoic acid glycosyltransferase protects *Staphylococcus aureus* from the lytic activity of Podoviridae. *Sci. Rep.* **5**, 17219 (2015).

35. K. Kurokawa, D. J. Jung, J. H. An, K. Fuchs, Y. J. Jeon, N. H. Kim, X. Li, K. Tateishi, J. A. Park, G. Xia, M. Matsushita, K. Takahashi, H. J. Park, A. Peschel, B. L. Lee, Glycoepitopes of staphylococcal

wall teichoic acid govern complement-mediated opsonophagocytosis via human serum antibody and mannose-binding lectin. *J. Biol. Chem.* **288**, 30956–30968 (2013).

36. T. C. Meyer, S. Michalik, S. Holtfreter, S. Weiss, N. Friedrich, H. Völzke, T. Kocher, C. Kohler, F. Schmidt, B. M. Bröker, U. Völker, A comprehensive view on the human antibody repertoire against *Staphylococcus aureus* antigens in the general population. *Front. Immunol.* **12**, 651619 (2021).

37. H. K. Kim, V. Thammavongsa, O. Schneewind, D. Missiakas, Recurrent infections and immune evasion strategies of *Staphylococcus aureus*. *Curr. Opin. Microbiol.* **15**, 92–99 (2012).

38. R. van Dalen, M. M. Molendijk, S. Ali, K. P. M. van Kessel, P. Aerts, J. A. G. van Strijp, C. J. C. de Haas, J. Codée, N. M. van Sorge, Do not discard *Staphylococcus aureus* WTA as a vaccine antigen. *Nature* **572**, E1-E2 (2019).

39. D. Gerlach, R. N. Sieber, J. Larsen, J. Krusche, C. de Castro, J. Baumann, A. Molinaro, A. Peschel, Horizontal transfer and phylogenetic distribution of the immune evasion factor *tarP*. *Front. Microbiol.* **13**, 951333 (2022).

40. L. Fišarová, T. Botka, X. Du, I. Mašlaňová, P. Bárdy, R. Pantůček, M. Benešík, P. Roudnický, V. Winstel, J. Larsen, R. Rosenstein, A. Peschel, J. Doškař, *Staphylococcus epidermidis* phages transduce antimicrobial resistance plasmids and mobilize chromosomal islands. *mSphere* **6**, e00223–21 (2021).

41. V. Winstel, C. Liang, P. Sanchez-Carballo, M. Steglich, M. Munar, B. M. Bröker, J. R. Penadés, U. Nübel, O. Holst, T. Dandekar, A. Peschel, G. Xia, Wall teichoic acid structure governs horizontal gene transfer between major bacterial pathogens. *Nat. Commun.* **4**, 2345 (2013).

42. J. L. Morgan, J. Strumillo, J. Zimmer, Crystallographic snapshot of cellulose synthesis and membrane translocation. *Nature* **493**, 181–186 (2013).

43. L. L. Lairson, B. Henrissat, G. J. Davies, S. G. Withers, Glycosyltransferases: Structures, functions, and mechanisms. *Annu. Rev. Biochem.* **77**, 521–555 (2008).

44. C. Breton, S. Fournel-Gigleux, M. M. Palcic, Recent structures, evolution and mechanisms of glycosyltransferases. *Curr. Opin. Struct. Biol.* **22**, 540–549 (2012).

45. S. Sobhanifar, L. J. Worrall, D. T. King, G. A. Wasney, L. Baumann, R. T. Gale, M. Nosella, E. D. Brown, S. G. Withers, N. C. J. Strynadka, Structure and mechanism of *Staphylococcus aureus* TarS, the wall teichoic acid β-glycosyltransferase involved in methicillin resistance. *PLoS Pathog.* **12**, e1006067 (2016).

46. L. Yakovlieva, M. T. C. Walvoort, Processivity in bacterial glycosyltransferases. *ACS Chem. Biol.* **15**, 3–16 (2020).

47. E. Vinogradov, I. Sadovskaya, J. Li, S. Jabbouri, Structural elucidation of the extracellular and cell-wall teichoic acids of *Staphylococcus aureus* MN8m, a biofilm forming strain. *Carbohydr. Res.* **341**, 738–743 (2006).

48. S. I. Morse, Studies on the chemistry and immunochemistry of cell walls of *Staphylococcus aureus*. *J. Exp. Med.* **116**, 229–245 (1962).

49. S. G. Nathenson, N. Ishimoto, J. S. Anderson, J. L. Strominger, Enzymatic synthesis and immunochemistry of α- and β-N-acetylglucosaminylribitol linkages in teichoic acids from several strains of *Staphylococcus aureus*. *J. Biol. Chem.* **241**, 651–658 (1966).

50. P. A. Driguez *et al.*, Immunogenic compositions against *S. aureus*. WO Patent WO/2017/064190 (2017).

51. S. Kilcher, M. J. Loessner, Engineering bacteriophages as versatile biologics. *Trends Microbiol.* **27**, 355–367 (2019).

52. V. Winstel, P. Kuhner, H. Rohde, A. Peschel, Genetic engineering of untransformable coagulase-negative staphylococcal pathogens. *Nat. Protoc.* **11**, 949–959 (2016).

53. S. M. Lehar, T. Pillow, M. Xu, L. Staben, K. K. Kajihara, R. Vandlen, L. DePalatis, H. Raab, W. L. Hazenbos, J. Hiroshi Morisaki, J. Kim, S. Park, M. Darwish, B. C. Lee, H. Hernandez, K. M. Loyet, P. Lupardus, R. Fong, D. Yan, C. Chalouni, E. Luis, Y. Khalfin, E. Plise, J. Cheong, J. P. Lyssikatos, M. Strandh, K. Koefoed, P. S. Andersen, J. A. Flygare, M. Wah Tan, E. J. Brown, S. Mariathasan, Novel antibody-antibiotic conjugate eliminates intracellular *S. aureus*. *Nature* **527**, 323–328 (2015).

54. A. Hendriks, R. van Dalen, S. Ali, D. Gerlach, G. A. van der Marel, F. F. Fuchsberger, P. C. Aerts, C. J. C. de Haas, A. Peschel, C. Rademacher, J. A. G. van Strijp, J. D. C. Codée, N. M. van Sorge, Impact of glycan linkage to *Staphylococcus aureus* wall teichoic acid on langerin recognition and langerhans cell activation. *ACS Infect. Dis.* **7**, 624–635 (2021).

55. C. Weidenmaier, J. F. Kokai-Kun, S. A. Kristian, T. Chanturiya, H. Kalbacher, M. Gross, G. Nicholson, B. Neumeister, J. J. Mond, A. Peschel, Role of teichoic acids in *Staphylococcus aureus* nasal colonization, a major risk factor in nosocomial infections. *Nat. Med.* **10**, 243–245 (2004).

56. S. Ali, A. Hendriks, R. van Dalen, T. Bruyning, N. Meeuwenoord, H. S. Overkleeft, D. V. Filippov, G. A. van der Marel, N. M. van Sorge, J. D. C. Codée, (Automated) Synthesis of well-defined *Staphylococcus aureus* wall teichoic acid fragments. *Chemistry* **27**, 10461–10469 (2021).

57. W. Kabsch, Integration, scaling, space-group assignment and post-refinement. *Acta Crystallogr. D Biol. Crystallogr.* **66**, 133–144 (2010).

58. A. J. Mc Coy, R. W. Grosse-Kunstleve, P. D. Adams, M. D. Winn, L. C. Storoni, R. J. Read, Phaser crystallographic software. *J. Appl. Crystallogr.* **40**, 658–674 (2007).

59. N. Stein, CHAINSAW: A program for mutating pdb files used as templates in molecular replacement. *J. Appl. Crystallogr.* **41**, 641–643 (2008).

60. P. Emsley, B. Lohkamp, W. G. Scott, K. Cowtan, Features and development of Coot. *Acta Crystallogr. D Biol. Crystallogr.* **66**, 486–501 (2010).

61. G. N. Murshudov, P. Skubák, A. A. Lebedev, N. S. Pannu, R. A. Steiner, R. A. Nicholls, M. D. Winn, F. Long, A. A. Vagin, REFMAC5 for the refinement of macromolecular crystal structures. *Acta Crystallogr. D Biol. Crystallogr.* **67**, 355–367 (2011).

62. O. S. Smart, T. O. Womack, C. Flensburg, P. Keller, W. Paciorek, A. Sharff, C. Vonrhein, G. Bricogne, Exploiting structure similarity in refinement: Automated NCS and target-structure restraints in BUSTER. *Acta Crystallogr. D Biol. Crystallogr.* **68**, 368–380 (2012).

63. P. D. Adams, P. V. Afonine, G. Bunkóczi, V. B. Chen, I. W. Davis, N. Echols, J. J. Headd, L. W. Hung, G. J. Kapral, R. W. Grosse-Kunstleve, A. J. McCoy, N. W. Moriarty, R. Oeffner, R. J. Read, D. C. Richardson, J. S. Richardson, T. C. Terwilliger, P. H. Zwart, PHENIX: A comprehensive Python-based system for macromolecular structure solution. *Acta Crystallogr. D Biol. Crystallogr.* **66**, 213–221 (2010).

64. A. W. Schuttelkopf, D. M. van Aalten, PRODRG: A tool for high-throughput crystallography of protein-ligand complexes. *Acta Crystallogr. D Biol. Crystallogr.* **60**, 1355–1363 (2004).

65. V. B. Chen, W. B. Arendall III, J. J. Headd, D. A. Keedy, R. M. Immormino, G. J. Kapral, L. W. Murray, J. S. Richardson, D. C. Richardson, MolProbity: All-atom structure validation for macromolecular crystallography. *Acta Crystallogr. D Biol. Crystallogr.* **66**, 12–21 (2010).

66. L. L. C. Schrodinger, *The PyMOL Molecular Graphics System Version* 1.8 (2015).

67. F. Madeira, M. Pearce, A. R. N. Tivey, P. Basutkar, J. Lee, O. Edbali, N. Madhusoodanan, A. Kolesnikov, R. Lopez, Search and sequence analysis tools services from EMBL-EBI in 2022. *Nucleic Acids Res.* (2022) **50**, W276–W279(2022).
